# Activation of innate immune receptor TLR9 by mitochondrial DNA plays essential roles in the chemical long-term depression of hippocampal neurons

**DOI:** 10.1016/j.jbc.2024.105744

**Published:** 2024-02-13

**Authors:** Naoya Atarashi, Misaki Morishita, Shinji Matsuda

**Affiliations:** 1 Department of Engineering Science, Graduate School of Informatics and Engineering, The University of Electro-Communications, Tokyo, Japan; 2 Center for Neuroscience and Biomedical Engineering (CNBE), The University of Electro-Communications, Tokyo, Japan

**Keywords:** toll-like receptor, mitochondria, caspase-3, endocytosis, ionotropic glutamate receptor, neuron, synaptic plasticity, long-term depression, AMPA receptor

## Abstract

Synaptic plasticity is believed to be the cellular basis for experience-dependent learning and memory. Although long-term depression (LTD), a form of synaptic plasticity, is caused by the activity-dependent reduction of cell surface α-amino-3-hydroxy-5-methyl-4-isoxazole propionic acid (AMPA)-type glutamate receptors (AMPA receptors) at postsynaptic sites, its regulation by neuronal activity is not completely understood. In this study, we showed that the inhibition of toll-like receptor-9 (TLR9), an innate immune receptor, suppresses N-methyl-d-aspartate (NMDA)-induced reduction of cell surface AMPA receptors in cultured hippocampal neurons. We found that inhibition of TLR9 also blocked NMDA-induced activation of caspase-3, which plays an essential role in the induction of LTD. siRNA-based knockdown of TLR9 also suppressed the NMDA-induced reduction of cell surface AMPA receptors, although the scrambled RNA had no effect on the NMDA-induced trafficking of AMPA receptors. Overexpression of the siRNA-resistant form of TLR9 rescued the AMPA receptor trafficking abolished by siRNA. Furthermore, NMDA stimulation induced rapid mitochondrial morphological changes, mitophagy, and the binding of mitochondrial DNA (mtDNA) to TLR9. Treatment with dideoxycytidine and mitochondrial division inhibitor-1, which block mtDNA replication and mitophagy, respectively, inhibited NMDA-dependent AMPA receptor internalization. These results suggest that mitophagy induced by NMDA receptor activation releases mtDNA and activates TLR9, which plays an essential role in the trafficking of AMPA receptors during the induction of LTD.

The α-amino-3-hydroxy-5-methyl-4-isoxazole propionic acid (AMPA)-type glutamate receptor (AMPA receptor) mainly mediates fast excitatory neurotransmission in the vertebrate central nervous system (1). Alterations in neural activity induce long-lasting changes in synaptic transmission, such as long-term depression (LTD), which underlies certain aspects of learning and memory (2, 3). When N-methyl-d-aspartate (NMDA)-type glutamate receptors (NMDA receptors) are moderately activated by LTD-inducing stimuli, postsynaptic AMPA receptors are thought to be freed from their anchoring proteins, diffuse laterally to the endocytic zone, and undergo clathrin-dependent endocytosis (2, 4, 5). They are subsequently transported to the late endosome/lysosomes, reducing the amount of cell surface AMPA receptors in the long term (6).

The molecular mechanisms by which neuronal activity controls the intracellular trafficking of AMPA receptors have been actively investigated. Trafficking of AMPA receptors is controlled by phosphorylation and dephosphorylation of AMPA receptors and transmembrane AMPA receptor regulatory proteins. Activation of NMDA receptors changes the phosphorylation status of AMPA receptors and transmembrane AMPA receptor regulatory proteins, which controls the binding to the postsynaptic anchoring proteins as well as adaptor protein complexes controlling the intracellular trafficking of membrane proteins (3, 6, 7, 8, 9).

It has also been reported that NMDA receptor–dependent AMPA receptor endocytosis requires signals from mitochondria upon LTD induction. Recent studies have indicated that cytochrome c release from mitochondria triggers AMPA receptor endocytosis through a moderate and transient activation of caspase-3 *via* the BAD-BAX cascade (10, 11).

Furthermore, autophagy plays a crucial role in both the induction and maintenance phases of LTD (12, 13, 14, 15). The activation of NMDA receptors induces autophagy in the postsynaptic dendrites (13), degraded AMPA receptors (14), and PSD-95 (12), which plays essential roles in the postsynaptic localization of AMPA receptors (16). In addition, Pink1 and Parkin, which are essential for mitochondrial autophagic degradation (mitophagy), have also been reported to be required for the induction of LTD (17, 18).

Although these studies have advanced our understanding of the molecular mechanisms of LTD, the precise molecular mechanisms remain unclear. For example, it remains unclear whether mitophagy is induced during the LTD induction and how it is related to AMPA receptor endocytosis. Therefore, further research is necessary to elucidate these molecular mechanisms.

Recently, toll-like receptors (TLR) 7 and 9 have been shown to play an important role in the induction of neuronal apoptosis *via* the activation of caspase-3 (19). A study on the developing mouse brain has shown that TLR7 is transiently expressed in the developing brain, whereas TLR9 has been reported to be upregulated at postnatal day 0 and maintains high expression even after 5 months of age (20). This indicates that TLR9 is expressed in mature neurons and activates caspase-3, which is essential for the internalization of AMPA receptors during LTD induction. Moreover, TLR9 can be activated by mitochondrial DNA (mtDNA), which is released from mitochondria during the mitophagy (21, 22).

Interestingly, one of the downstream proteins of TLR9 is p38 mitogen-activated protein kinase (MAPK) (23), a crucial factor in the induction of NMDA-dependent LTD (24). In human keratinocytes, it has been reported that cytochrome c release is induced by the p38 MAPK-dependent BAX translocation to the mitochondria and this released cytochrome c subsequently activates caspase-3 during the apoptosis induction (25). This pathway may play a role in LTD induction; however, there have been no observations of BAX translocation to the mitochondria during the LTD induction process (10). Alternatively, p38 MAPK can activate caspase-3 by reducing the interaction between caspase-2 and procaspase-3 (26). Caspase-2 has also been shown to have essential roles in the internalization of AMPA receptors during the LTD induction (27).

Therefore, we hypothesized that mtDNA released from mitochondria by mitophagy activates TLR9 and plays a role in the LTD induction by activating caspase-3. We found that the inhibition and knockdown of TLR9, as well as the inhibition of mtDNA amplification, attenuated the NMDA-induced reduction of cell surface AMPA receptors, indicating that TLR9 activation by mtDNA is required for LTD induction.

## Results

### Inhibition of TLR9 blocked the NMDA-induced AMPA receptor endocytosis

**Figure 1:**
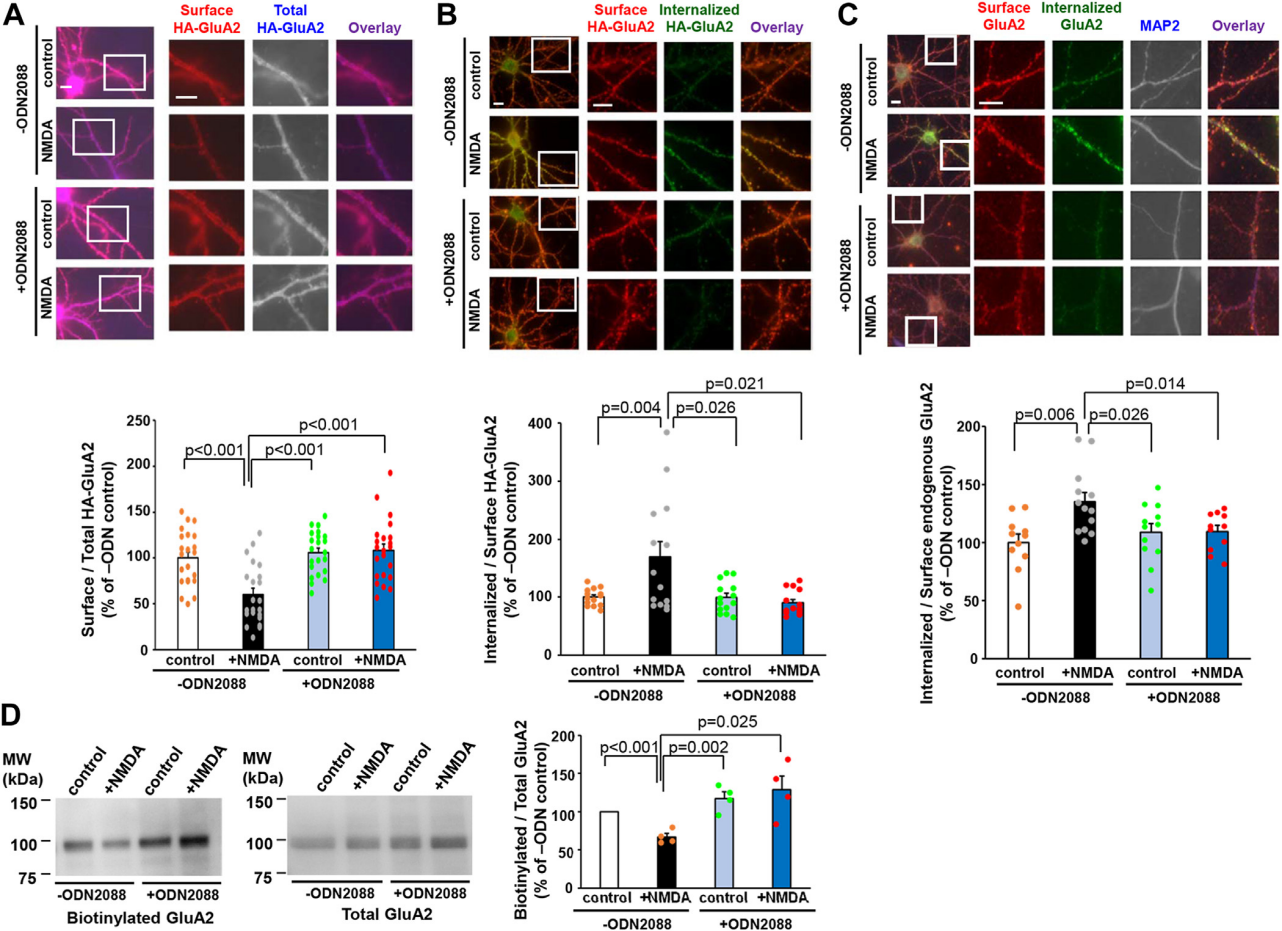
**ODN2088 blocks NMDA-induced internalization of GluA2 containing AMPA receptors.***A*, immunocytochemical analysis of the effects of ODN2088 on the NMDA-induced reduction of cell surface GluA2. Cultured hippocampal neurons expressing hemagglutinin (HA)-tagged GluA2 were treated with 50 μM NMDA for 10 min without or with ODN2088 (1 μM, 10 min). After fixation, cell surface HA-GluA2 were stained (*red*) and after treatment with Triton X-100, neurons were immunostained for total HA-GluA2 (*blue*). The dendritic regions marked by *squares* are enlarged in the panels to the *right*. The scale bar represents 10 μm. *Lower graph*: quantification of the NMDA-induced reduction in the ratio of surface to total HA-GluA2 fluorescence intensities. Data are represented as the ratio of surface HA-GluA2 immunoreactivity to total HA-GluA2 immunoreactivity. The ratio of control neurons without ODN2088 treatment was defined as 100% (n = 22). Data are presented as mean + SEMs and individual data points. *p* value by one-way ANOVA, followed by Student-Newman-Keuls post hoc test. *B* and *C*, antibody-feeding assay evaluating the effects of ODN2088 on NMDA-induced internalization of cell surface HA-GluA2 (*B*) and endogenous GluA2 (*C*). *B*, living neurons expressing exogenous HA-GluA2 were labeled with anti-HA antibodies. Neurons were treated with 50 μM NMDA for 10 min with or without ODN2088 treatment. After fixation, cell surface HA antibodies were stained (*red*), and after treatment with Triton X-100, internalized HA antibodies were stained (*green*). The dendritic regions marked by *squares* are magnified in the panels to the *right*. The scale bar represents 10 μm. *Lower graph*: quantification of the NMDA-induced increase in the ratio of internalized to surface HA-antibody fluorescence intensities. The ratio of control neurons without ODN2088 treatment was defined as 100% (n = 14). Data are presented as mean + SEM and individual data points. *p* value by one-way ANOVA followed by Student−Newman−Keuls post hoc test. *C*, endogenous GluA2 in living neurons was labeled with antibodies against the extracellular region of GluA2. Neurons were treated with 50 μM NMDA for 10 min with or without ODN2088 treatment. After fixation, cell surface GluA2 antibodies were stained (*red*), and after treatment with Triton X-100, internalized GluA2 antibodies (*green*) and the dendritic marker MAP2 were stained (*blue/white*). The dendritic regions marked by *squares* are enlarged in the panels to the *right*. The scale bar represents 10 μm. *Lower graph*: quantification of the NMDA-induced increase in the ratio of internalized to surface GluA2-antibody fluorescence intensities. The ratio of control neurons without ODN2088 treatment was defined as 100% (n = 11–13). Data are presented as mean + SEM and individual data points. *p* value by one-way ANOVA followed by Student-Newman-Keuls post hoc test. *D*, biotinylation assay of endogenous GluA2. Hippocampal cultures were stimulated by NMDA without or with ODN2088. Cell surface proteins were biotinylated and pulled down from the total cell lysates. The amount of GluA2 proteins in the pulled down fraction (*left gel*) and total cell lysate fraction (*right gel*) were analyzed with the immunoblot analysis. NMDA stimulation decreased the amount of the cell surface GluA2, and treatment with ODN2088 blocked the NMDA effect. *Right graph*: the intensity of the GluA2 band in the biotinylated fraction was normalized to that of the total cell lysate fraction. The ratio of biotinylated/total GluA2 in the control cultures without ODN2088 treatment was arbitrarily set to 100% (n = 4 each). Data are presented as mean + SEM and individual data points. *p* value by Kruskal–Wallis test and Student-Newman-Keuls post hoc test. AMPA, α-amino-3-hydroxy-5-methyl-4-isoxazole propionic acid; NMDA, N-methyl-d-aspartate.

To investigate the role of TLR9 in the induction of LTD, we inhibited TLR9 and analyzed AMPA receptor trafficking using a chemical LTD model. In this model, NMDA application induces AMPA receptor endocytosis. We expressed the AMPA receptor subunit GluA2, in which a hemagglutinin (HA) tag was added to the N-terminal extracellular domain (HA-GluA2), in cultured hippocampal neurons. After treatment with NMDA (50 μM) for 10 min with or without pretreatment with a TLR9 antagonist (ODN 2088), the cell surface and total HA-GluA2 were sequentially detected using an anti-HA antibody before and after permeabilization of the plasma membrane. As expected, NMDA treatment reduced the intensity of cell surface HA-GluA2 in cultured hippocampal neurons without ODN2088 treatment (Fig. 1*A*; *p* < 0.001, one-way ANOVA followed by Student-Newman-Keuls post hoc test, n = 22 cells from three independent cultures prepared on different days).

While the treatment with ODN2088 did not affect the amount of cell surface HA-GluA2 under control conditions (*p* = 0.919, n = 22 cell), the intensity of cell surface HA-GluA2 was not reduced by NMDA treatment in the ODN2088-treated neurons (*p* = 0.82, n = 22 cells). These results suggested that TLR9 activity is necessary for NMDA-induced endocytosis of GluA2. We also analyzed the effect of ODN2088 on NMDA-induced endocytosis of the HA-tagged GluA1 subunit (HA-GluA1). The NMDA treatment resulted in a reduction in cell surface HA-GluA1 levels in the absence of ODN2088 (Fig. S1*A*; *p* = 0.037, one-way ANOVA followed by Student-Newman-Keuls post hoc test, n = 15 cells from two independent cultures prepared on different days). However, the amplitude of the reduction in cell surface GluA1 was smaller than that of GluA2. In the presence of ODN2088, NMDA had no effect on the amount of cell surface HA-GluA1 (*p* = 0.639 n = 15 cells). These results indicated that the endocytosis of GluA1 is partially induced by the NMDA and this partial endocytosis requires the activation of TLR9.

To examine whether ODN2088 had any impact on the function of NMDA receptor, we used a chemical long-term potentiation (LTP) model in which the application of glycine (Gly), a coagonist of the NMDA receptors, induces AMPA receptor exocytosis (28). We expressed HA-GluA1 in cultured hippocampal neurons and stimulated neurons with Gly (200 μM) in the presence or absence of ODN2088. After inducing chemical LTP, cell surface and total HA-GluA1 were sequentially detected using an anti-HA antibody and the ratio of cell surface to total HA-GluA1 was quantified (Fig. S1*B*). Gly treatment led to an increase in the cell surface HA-GluA1 intensity in cultured hippocampal neurons in both ODN2088-treated and ODN2088-untreated cultures, indicating that ODN2088 did not affect the NMDA-induced exocytosis of AMPA receptors (*p* < 0.001, Kruskal–Wallis test followed by Student-Newman-Keuls post hoc test, n = 21 cells from three independent cultures prepared on different days). These results suggested that the ODN2088 treatment had no effects on the function of NMDA receptors.

To further confirm the effect of ODN2088 on NMDA-induced endocytosis of AMPA receptors, we conducted an antibody-feeding assay. We applied anti-HA antibodies to the HA-GluA2–expressing neurons and stimulated them with NMDA in the presence or absence of ODN2088. We sequentially detected cell surface and internalized antibodies using Alexa 546–and Alexa 488–conjugated secondary antibodies before and after membrane permeabilization, respectively (Fig. 1*B*). NMDA treatment significantly increased the ratio of internalized/cell surface HA-GluA2 in the absence of ODN2088 (*p* = 0.004, n = 14 cells from two independent cultures prepared on different days, one-way ANOVA followed by Student-Newman-Keuls post hoc test). Conversely, NMDA stimulation failed to induce HA-GluA2 internalization in the presence of ODN2088. We also examined the internalization of endogenous GluA2 by using both the antibody-feeding assay and biotinylation assay. In the antibody-feeding assay, we applied antibodies against the N-terminal extracellular region of GluA2 to the neurons and stimulated them with NMDA (Fig. 1*C*). The amount of internalized GluA2 antibodies was significantly increased with NMDA stimulation in the absence of ODN2088 (*p* = 0.006, n = 11–13 cells from two independent cultures prepared on different days, one-way ANOVA followed by Student-Newman-Keuls post hoc test). However, ODN2088 treatment nullified the effect of NMDA stimulation (*p* = 0.937, n = 11–12 cells from two independent cultures prepared in different days). In the biotinylation assay, neurons were stimulated with NMDA in the presence or absence of ODN2088 treatment and the cell surface proteins were biotinylated and pulled down with avidin-conjugated beads. The amount of pulled down GluA2 protein and the amount of GluA2 protein in the total cell lysates were analyzed by immunoblot analysis (Fig. 1*D*). The ratio of biotinylated GluA2 to total GluA2 was significantly decreased with NMDA stimulation in the absence of ODN2088 (*p* < 0.001, Kruskal–Wallis test and Student-Newman-Keuls post hoc test, n = 4), although the ratio was not reduced in the presence of ODN2088 (*p* = 0.546, n = 4). We confirmed that the intracellular protein, actin, was not biotinylated in all conditions (Fig. S1*C*). These results confirmed that ODN2088 treatment blocked the NMDA-induced endocytosis of AMPA receptors.

### TLR9 knockdown blocked NMDA-induced AMPA receptor endocytosis

**Figure 2:**
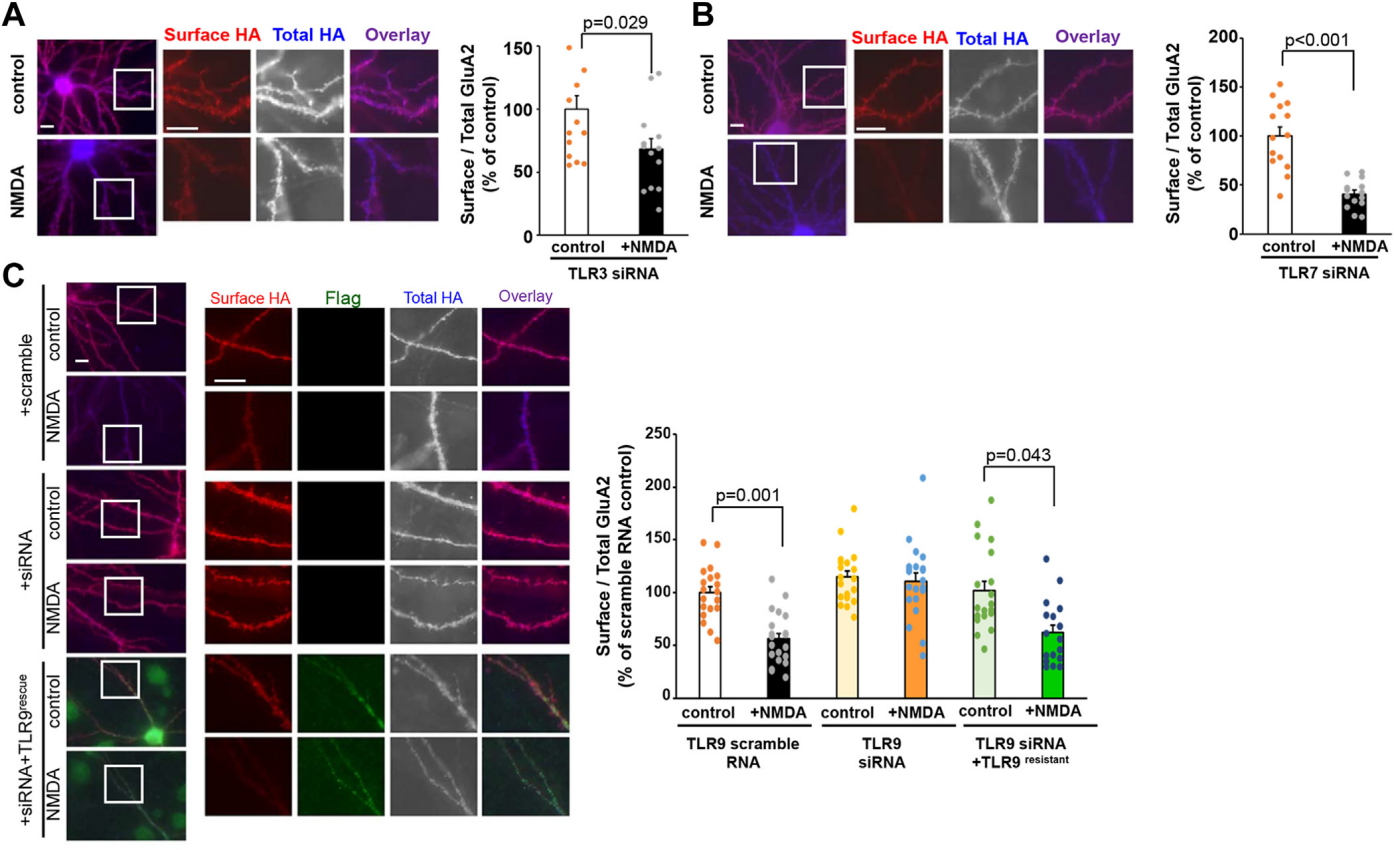
**TLRs function on NMDA-induced reduction of cell surface GluA2.***A* and *B*, immunocytochemical analysis of the effects of TLR3 knockdown (*A*) and TLR7 knockdown (*B*) on the NMDA-induced endocytosis of cell surface GluA2. Cultured hippocampal neurons expressing HA-tagged GluA2 with siRNA were treated with 50 μM NMDA for 10 min. Following fixation, the cell surface HA-GluA2 (*red*) and total HA-GluA2 (*blue*) were stained. The dendritic regions marked by *squares* were enlarged in the panels to the *right*. The scale bar represents 10 μm. *Right graph*: quantification of NMDA-induced reduction in the ratio of surface to total HA-GluA2 fluorescence intensity. Data are represented as the ratio of surface HA-GluA2 immunoreactivity normalized by total HA-GluA2 immunoreactivity. The ratio of control neurons was defined as 100% (n = 14). Data are presented as mean + SEM and individual data points. *p* value by two-tailed Student’s *t* test. *C*, immunocytochemical analysis of the effects of TLR9 knockdown on the NMDA-induced endocytosis of cell surface GluA2. Cultured hippocampal neurons expressing HA-tagged GluA2 with scrambled RNA (*top*), siRNA (*middle*), and siRNA with TLR9^resistant^-FL (*bottom*) were treated with 50 μM NMDA for 10 min. Following fixation, the cell surface HA-GluA2 (*red*), TLR9^resistant^-FL (*green*), and total HA-GluA2 (*blue*) were stained. The dendritic regions marked by *squares* were enlarged in the panels to the *right*. The scale bar represents 10 μm. *Right graph*: quantification of NMDA-induced reduction in the ratio of surface to total GluA2 fluorescence intensity. Data are represented as the ratio of surface HA-GluA2 immunoreactivity normalized by total HA-GluA2 immunoreactivity. The ratio of scramble RNA transfected control neurons was defined as 100% (n = 18–21). Data are presented as mean + SEM and individual data points. *p* value by Kruskal–Wallis test and Dunn’s post hoc test. HA, hemagglutinin; TLR, toll-like receptor; NMDA, N-methyl-d-aspartate.

Since ODN2088 is recognized for its ability to inhibit the function of TLR3, TLR7/8, and TLR9, we sought to identify the TLRs crucial for LTD induction. First, we examined the expression of these proteins in our hippocampal cultures by using immunocytochemical analysis. The results indicated that the TLR3, TLR7/8, and TLR9 were all expressed in the MAP2-positive neurons (Fig. S2*A*). To identify which TLRs are essential for the induction of NMDA-induced LTD, we knocked down TLR3, TLR7, and TLR9 by using predesigned siRNA purchased from Sigma-Aldrich. The siRNAs for TLRs were transfected into cultured hippocampal neurons along with an enhanced GFP expression vector, and the amount of endogenous TLR proteins was quantified using immunocytochemical analysis (Fig. S2, *B* and *E*). Transfection of siRNA into hippocampal neurons resulted in the endogenous TLRs becoming barely detectable through immunohistochemistry. The intensity of TLR immunoreactivity was significantly reduced in siRNA-transfected neurons compared with neighboring neurons (Figs. S2, *C* and *D*, and S3*F*). In the TLR3 and TLR7 knocked down neurons, NMDA treatment significantly reduced the amount of cell surface HA-GluA2 (Fig. 2, *A* and *B* *p* = 0.029, n = 14–15 cells from two independent cultures, and *p* < 0.001, n = 14 cells from two independent cultures, respectively, two-tailed Student’s *t* test). Conversely, NMDA treatment did not induce a reduction in cell surface HA-GluA2 immunoreactivity in neurons transfected with the siRNA against TLR9 (Fig. 2*C* middle panels). We confirmed that transfection of scramble RNA, which had no effects on the expression level of TLR9 (Fig. S2, *E* and *F*), did not block the NMDA-induced reduction of cell surface HA-GluA2 (Fig. 2*C* top panels; *p* = 0.001, n = 19–21 cells from three independent cultures prepared on different days, Kruskal–Wallis test and Student-Newman-Keuls post hoc test). No difference was observed in the ratio of surface to total HA-GluA2 under control conditions between neurons transfected with scramble RNA and siRNA-transfected neurons (*p* = 1.00). To rule out the off-target effect of siRNA, we introduced silent mutations in the complementary DNA (cDNA) encoding the C-terminal Flag-tagged TLR9 to ensure that the encoded mRNA was refractory to siRNA-mediated knockdown (TLR9^resistant^-Flag). We introduced the expression vector for TLR9^resistant^-Flag into human embryonic kidney 293 (HEK293) cells with siRNA for TLR9. Immunoblot analysis indicated that cotransfection with siRNA did not reduce the expression of TLR9^resistant^-Flag (Fig. S2*G* left gel), whereas it almost completely blocked the expression of C-terminal FLAG-tagged WT TLR9, without affecting the expression of actin (Fig. S2*G* right gel). Cultured hippocampal neurons were transfected with TLR9 siRNA and cDNAs encoding HA-GluA2 and TLR9^resistant^-Flag and then treated neurons with NMDA for 10 min. The results indicated that NMDA treatment significantly reduced the amount of cell surface HA-GluA2 (Fig. 2*C*; *p* = 0.043). The ratio of surface to total HA-GluA2 under control conditions in TLR9 ^resistant^-Flag–expressing neurons was not different from those in the scramble and siRNA-transfected neurons (*p* = 1.00, n = 19 cells from three independent cultures). We also examined whether the overexpression of FLAG-tagged WT TLR9 could rescue the siRNA effects on the endocytosis of HA-GluA2. As shown in Fig. S3, NMDA stimulation did not induce a significant reduction of surface HA-GluA2 (*p* = 0.347, n = 11 cells from two independent cultures, two-tailed Student’s *t* test). These results confirm that TLR9 is essential for NMDA-induced endocytosis of AMPA receptors during the induction of chemical LTD.

### Morphology of mitochondria was affected by NMDA stimulation in hippocampal neurons

**Figure 3:**
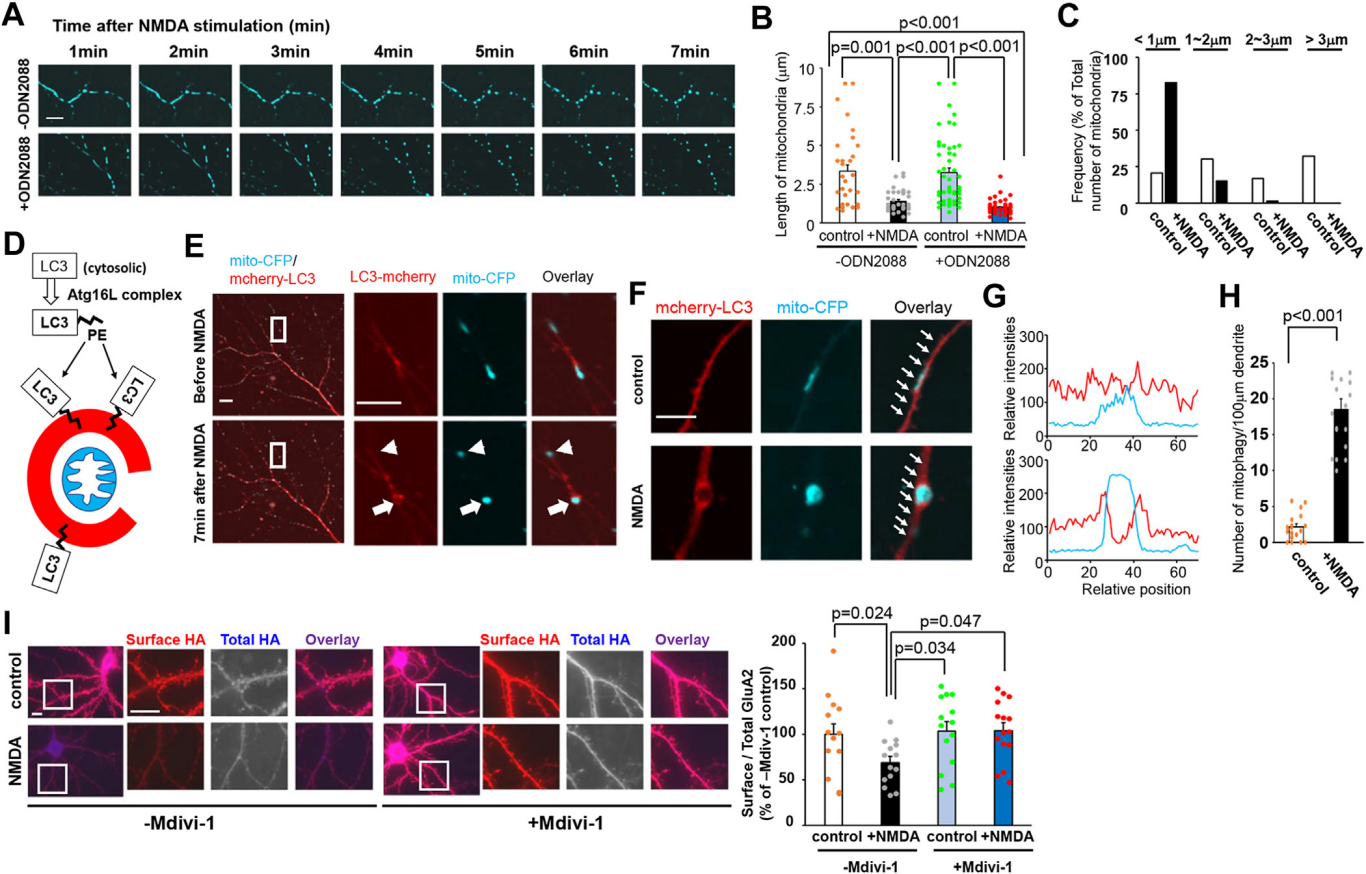
**Mitochondrial morphological changes and mitophagy induced by NMDA treatment.***A*, cultured hippocampal neurons expressing mitochondria-targeted cyan fluorescent protein (mito-CFP) were stimulated with NMDA and observed for up to 7 min. Images of the mitochondria every 1 min after NMDA stimulation from the ODN2088 untreated neuron (*upper panel*) and ODN2088 treated neuron (*lower panel*). The scale bar represents 10 μm. *B*, quantitative analysis of the length of mitochondria. Cultured hippocampal neurons expressing mito-CFP were stimulated with NMDA for 7 min in the absence or presence of ODN2088. After fixation, the length of mitochondria was quantified. n = 37 to 50 mitochondria from three independent cultures. Data are presented as mean + SEM and individual data points. *p* value by Kruskal–Wallis test and Dunn’s post hoc test. *C*, histogram of mitochondrial length in the ODN2088 untreated neurons. *White* and *black bars* indicate the frequency of mitochondria without (control) or with the NMDA stimulation, respectively. *D*, schematic drawing of lipidation and translocation of microtubule-associated protein 1 light chain 3 (LC3) from the cytosol to the isolation membrane upon autophagy induction. Phosphatidylethanolamine is attached to cytosolic LC3 by the Atg16L complex and lipidated LC3 translocates to the isolation membrane of the autophagosome (21, 25). *E*, cultured hippocampal neurons expressing mito-CFP and mCherry-LC3B were stimulated with NMDA and observed for up to 7 min. Images of the neurons before and 7 min after NMDA stimulation are shown. The dendritic regions enclosed by the *white squares* are magnified in the *right panels*. The scale bars represent 10 μm in the *left panels* and 5 μm in the *right panels*. *Arrows* indicate the mitochondria surrounded by mCherry-LC3B. *Arrowheads* indicate mitochondria outside autophagosomes. *F*, high-resolution images of mCherry-LC3 and mito-CFP from the NMDA-untreated (control) and NMDA-stimulated neurons. The scale bars represent 5 μm. *G*, line scan of the fluorescence intensities of mCherry-LC3 and mito-CFP. The mCherry-LC3 (*red*) and mito-CFP (*cyan*) fluorescence were quantified along the dendrites indicated by *white arrows* in (F), indicating that the mito-CFP signal was surrounded by the mCherry-LC3 signal in the NMDA-stimulated neuron, whereas, mCherry-LC3 uniformly distributed along the dendrite in the control neuron. *H*, quantitative analysis of the number of mitophagy within the 100 μm dendrite. Data are presented as mean + SEM and individual data points. n = 18 from three independent cultures. *p* value by two-tailed Student’s *t* test. *I*, cultured hippocampal neurons expressing HA-GluA2 were pretreated with Mdivi-1 and stimulated with NMDA. The cell surface HA-GluA2 (*red*) and total HA-GluA2 (*blue*) were stained. The dendritic regions marked by *squares* were enlarged in the panels to the *right*. The scale bars represent 10 μm. *Right graph*: quantification of NMDA-induced reduction in the ratio of the surface to total GluA2 fluorescence intensities. Data are represented as the ratio of surface HA-GluA2 immunoreactivity normalized by total HA-GluA2 immunoreactivity. The ratio in Mdivi-1–untreated control neurons was defined as 100% (n = 14–15). Data are presented as mean + SEM and individual data points. ∗*p* < 0.05 by one-way ANOVA and Student-Newman-Keuls post hoc test. HA, hemagglutinin; Mdivi, mitochondrial division inhibitor; NMDA, N-methyl-d-aspartate.

We then examined how TLR9 is activated during LTD induction. The endogenous ligand of TLR9 is mtDNA, which contains an unmethylated CpG sequence (29). Moreover, it has been reported that the mitochondrial pathway is required for caspase-3 activation during the induction of LTD (11), and that mitochondrial morphology is dynamically changed during the induction of LTP, another form of synaptic plasticity (30). Therefore, we first examined the morphology of mitochondria after LTD-inducing stimulation (treatment with NMDA) using live imaging. Cultured hippocampal neurons expressing cyan fluorescent protein (CFP) with a mitochondria-targeting signal (mito-CFP) and mCherry were treated with NMDA, and the morphology of mitochondria (CFP) and dendrite (mCherry) was analyzed by live imaging. Fig. S4 shows the morphology of dendrites and mitochondria before and 7 min after NMDA treatment. Before treatment, mitochondria in the dendrites were elongated, whereas 7 min after treatment, they were rounded. However, the overall morphology of the dendrites was not affected by NMDA treatment. Similar mitochondrial morphological changes were observed in five independent cultured cells by live imaging. Time-lapse imaging showed that mitochondria shrinkage started at 3 min and ended at 5 min after NMDA treatment (Fig. 3*A* upper panel and Movie S1). We also examined whether the TLR9 activities are required for the morphological changes of mitochondria by carrying out the identical live imaging in the presence of ODN2088, indicating that the NMDA induced the morphological changes of mitochondria even in the presence of ODN2088 (Fig. 3*A* lower panel). We quantitatively analyzed the length of mitochondria using the fixed neurons. The results indicated that the length of mitochondria was significantly reduced by the NMDA treatment both in the ODN-untreated and ODN-treated conditions (Fig. 3*B*; *p* = 0.001, n = 37–38 cells and *p* < 0.001, n = 50 cells, respectively from five independent cultures prepared on different days. Kruskal–Wallis test and Dunn’s post hoc test). ODN2088 treatment did not affect the length of mitochondria both in control and NMDA-stimulated neurons (*p* = 0.697, Kruskal–Wallis test and Dunn’s post hoc test). The histogram of the mitochondrial length further confirmed that the NMDA-stimulation induced the mitochondrial morphological changes (Fig. 3*C*). These results indicate that mitochondria shrink rapidly after NMDA stimulation without affecting the overall morphology of the dendrites and that the TLR9 contributes to the induction of LTD at downstream of mitochondrial morphological changes.

We also examined the reversibility of mitochondrial morphology changes (Fig. S5, *A* and *B*). Cultured hippocampal neurons expressing mito-CFP were subjected to a 7-min NMDA treatment. Following the removal of NMDA, neurons were incubated for 2 h and mitochondrial morphology was compared with that of control neurons and neurons treated with NMDA for 7 min. NMDA treatment significantly induced mitochondrial shrinkage, and 2 h of incubation after NMDA washout restored mitochondrial morphology. These results indicate that NMDA-stimulated mitochondrial shrinkage is a reversible process.

### Mitophagy induced by the NMDA stimulation was required for LTD induction

Next, we aimed to determine the physiological significance of NMDA-induced mitochondrial morphological changes. It is known that mitochondria can be degraded by autophagosomes under certain conditions, a phenomenon specifically termed mitophagy (31) and that the mtDNA can be released from mitochondria through mitophagy (32). Additionally, it has also been reported that autophagy is triggered during LTD induction (13). Therefore, we analyzed whether mitophagy occurs during LTD induction. As fluorescent protein-tagged microtubule-associated protein 1 light chain 3 (LC3) is widely used to visualize autophagy, including mitophagy (33, 34, 35), we generated an expression vector for N-terminal mCherry-tagged LC3B (mCherry-LC3B). During mitophagy induction, phosphatidylethanolamine attaches to cytosolic LC3 by the Atg16L complex and causes LC3 to translocate from the cytosol to the isolation membranes that surround the target mitochondria (31, 36) (Fig. 3*D*). To examine whether the shrunken mitochondria were digested by mitophagy, we coexpressed mito-CFP and mCherry-LC3B in cultured hippocampal neurons and performed live imaging (Fig. 3*E*). Before NMDA stimulation, mitochondria had an elongated morphology and LC3B did not surround the mitochondria. Seven minutes after NMDA stimulation, some of the shrunken mitochondria were surrounded by mCherry-LC3B–positive structures. Similar NMDA-induced mitophagy was observed in five independent cultured cells by live imaging. We further analyzed the NMDA-induced mitophagy by using fixed neurons at a higher resolution. Under control conditions, mCherry-LC3B was evenly distributed along the dendrite (Fig. 3*F*). Line scan analysis also confirmed the uniform distribution of mCherry-LC3B (Fig. 3*G*; red line). In neurons stimulated with NMDA for 7 min, mitochondria with a round shape were surrounded by mCherry-LC3B. We quantified the number of mitochondria surrounded by mCherry-LC3B along the dendrites (Fig. 3*H*). The number of mitophagy events in the dendrites significantly increased following NMDA stimulation (*p* < 0.001, n = 18 from three independent cultures prepared on different days, two-tailed Student’s *t* test). These results indicate that some parts of mitochondria were incorporated into autophagosomes after NMDA stimulation.

To examine the functional importance of mitophagy, hippocampal neurons were treated with the mitochondrial division inhibitor-1 (Mdivi-1), which is known to inhibit mitophagy (37). We first confirmed the inhibitory effect of Mdivi-1 treatment on mitophagy (Fig. S6). Neurons expressing mito-CFP and mCherry-LC3B were pretreated with Mdivi-1 (10 μM for 2 h), and we examined whether NMDA stimulation induced mitophagy or not. The results indicated that almost no mitophagy was observed, even in the NMDA-treated neurons. These findings suggest that Mdivi-1 effectively blocked mitophagy under our experimental conditions. Subsequently, we pretreated neurons expressing HA-GluA2 with Mdivi-1, and the ratio of surface to total HA-GluA2 was examined with or without NMDA stimulation. (Fig. 3*I*). As expected, in the absence of Mdivi-1, NMDA stimulation significantly reduced the surface/total HA-GluA2 ratio (*p* = 0.024, n = 14–15 cells from two independent cultures prepared on different days, one-way ANOVA and Student-Newman-Keuls post hoc test). Conversely, in neurons treated with Mdivi-1, NMDA stimulation did not lead to a reduction in the surface/total HA-GluA2 ratio (*p* = 0.952), indicating that mitophagy is required for the induction of NMDA-LTD.

### The mtDNA nucleoid size is key for LTD induction

**Figure 4:**
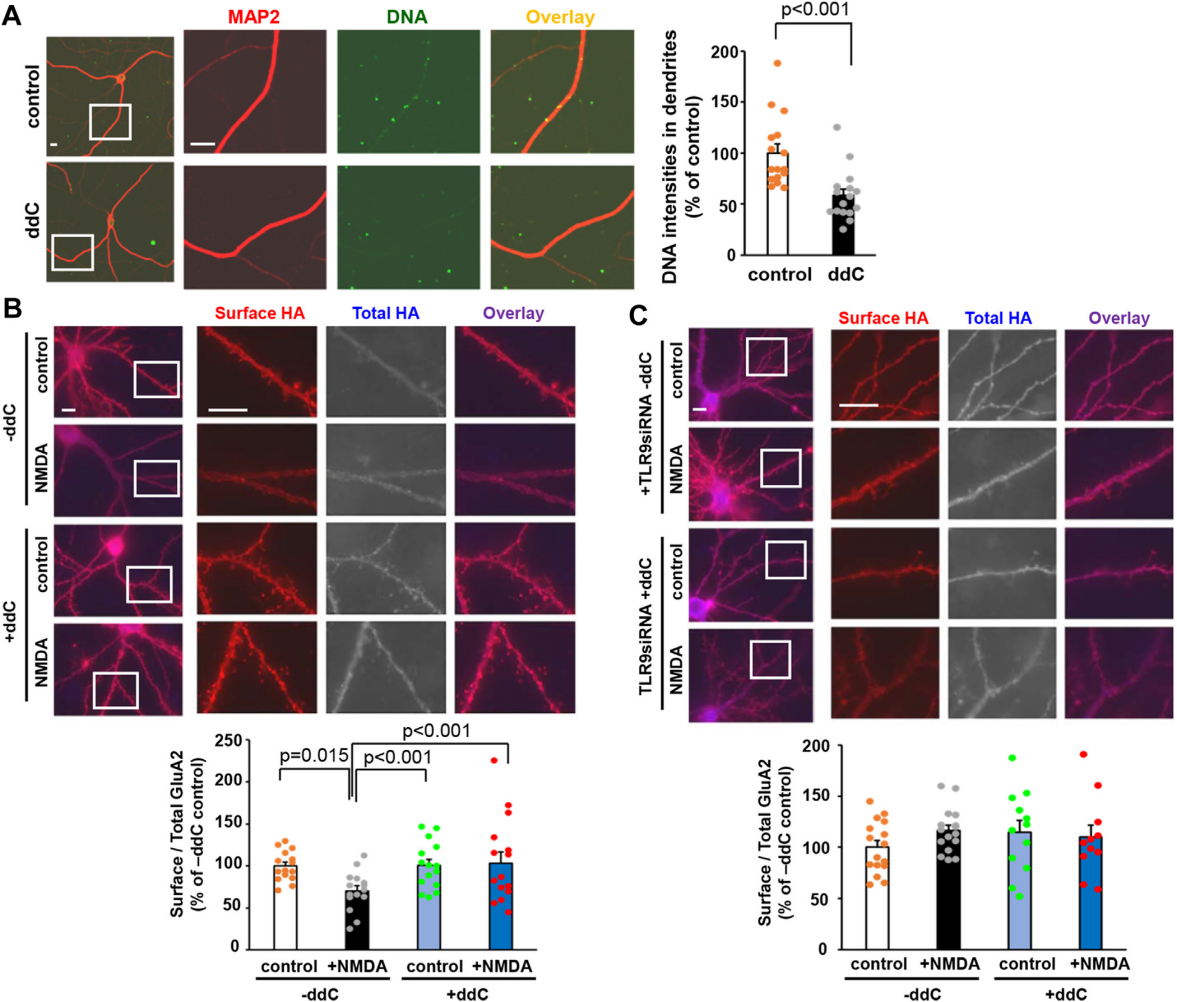
**Mitochondrial DNA amount affected the NMDA-induced internalization of AMPA receptors.***A*, dideoxycytidine (ddC) reduced the amount of mitochondrial DNA (mtDNA). Cultured hippocampal neurons were treated with ddC for 96 h and stained by the antiMAP2 and DNA antibodies. The fluorescence intensities of DNA staining within the dendrites were quantified. The average fluorescence intensities of ddC untreated (control) neurons were defined as 100% (n = 16). Data are presented as mean + SEM and individual data points. *p* value by two-tailed Student’s *t* test. The scale bars represent 10 μm. *B*, cultured hippocampal neurons expressing HA-GluA2 were pretreated with ddC and stimulated with NMDA. The cell surface HA-GluA2 (*red*) and total HA-GluA2 (*blue*) were stained. The dendritic regions marked by *squares* were enlarged in the panels to the *right*. The scale bars represent 10 μm. *Lower graph*: quantification of NMDA-induced reduction in the ratio of the surface to total GluA2 fluorescence intensities. Data are represented as the ratio of surface HA-GluA2 immunoreactivity normalized by total HA-GluA2 immunoreactivity. The ratio in ddC-untreated control neurons was defined as 100% (n = 15). Data are presented as mean + SEM and individual data points. *p* value by Kruskal–Wallis test and Student-Newman-Keuls post hoc test. *C*, ddC effects on the NMDA-induced AMPA receptor endocytosis in the TLR9 knocked down neurons. The ratio in ddC-untreated control neurons was defined as 100% (n = 16). Data are presented as mean + SEM and individual data points. No significant difference was detected by one-way ANOVA. The scale bars represent 10 μm. AMPA, α-amino-3-hydroxy-5-methyl-4-isoxazole propionic acid; HA, hemagglutinin; NMDA, N-methyl-d-aspartate; TLR, toll-like receptor.

We next investigated the role of mtDNA in the induction of LTD. This is because mitophagy is recognized to activate TLR9 through the release of mtDNA (32). Dideoxycytidine (ddC), which specifically inhibits mtDNA replication and reduces mtDNA nucleoid size, has been reported to drastically alleviate mtDNA stress in mouse embryonic fibroblasts (38). We first examined whether ddC treatment can reduce mtDNA levels in neurons. Cultured hippocampal neurons were treated with ddC for 96 h, and the amount of DNA was quantified using immunocytochemical analysis (Fig. 4*A*). The results revealed a significant reduction in DNA staining within the dendritic region of ddC-treated neurons compared to control neurons (*p* < 0.001, n = 16 from two independent cultures prepared on different days, two-tailed Student’s *t* test). We additionally validated that mitochondrial morphological changes were not inhibited by ddC treatment (Fig. S7). To examine the possibility that mtDNA functions as a signaling molecule to induce AMPA receptor endocytosis, we pretreated cultured hippocampal neurons expressing HA-GluA2 with ddC. In the absence of ddC treatment, NMDA stimulation significantly reduced the cell surface HA-GluA2 (Fig. 4*B*; *p* = 0.015, n = 15 from two independent cultures prepared on different days, Kruskal–Wallis test and Student-Newman-Keuls post hoc test). Conversely, in the ddC-treated neurons, NMDA treatment did not reduce cell surface HA-GluA2 (*p* = 0.94), indicating that mtDNA plays an essential role in inducing NMDA-dependent AMPA receptor endocytosis.

To explore the relationship between TLR9 and ddC, we pretreated TLR9 knocked down neurons with ddC. In both ddC-treated and untreated neurons, NMDA failed to induce a reduction in cell surface HA-GluA2. No additional effect was observed in the ddC-treated neurons (Fig. 4*C*; *p* = 0.456, n = 11–16 cells from two independent cultures prepared on different days, one-way ANOVA). Considering these results, one possibility is that mtDNA and TLR9 regulate AMPA receptor trafficking through a shared pathway.

**Figure 5:**
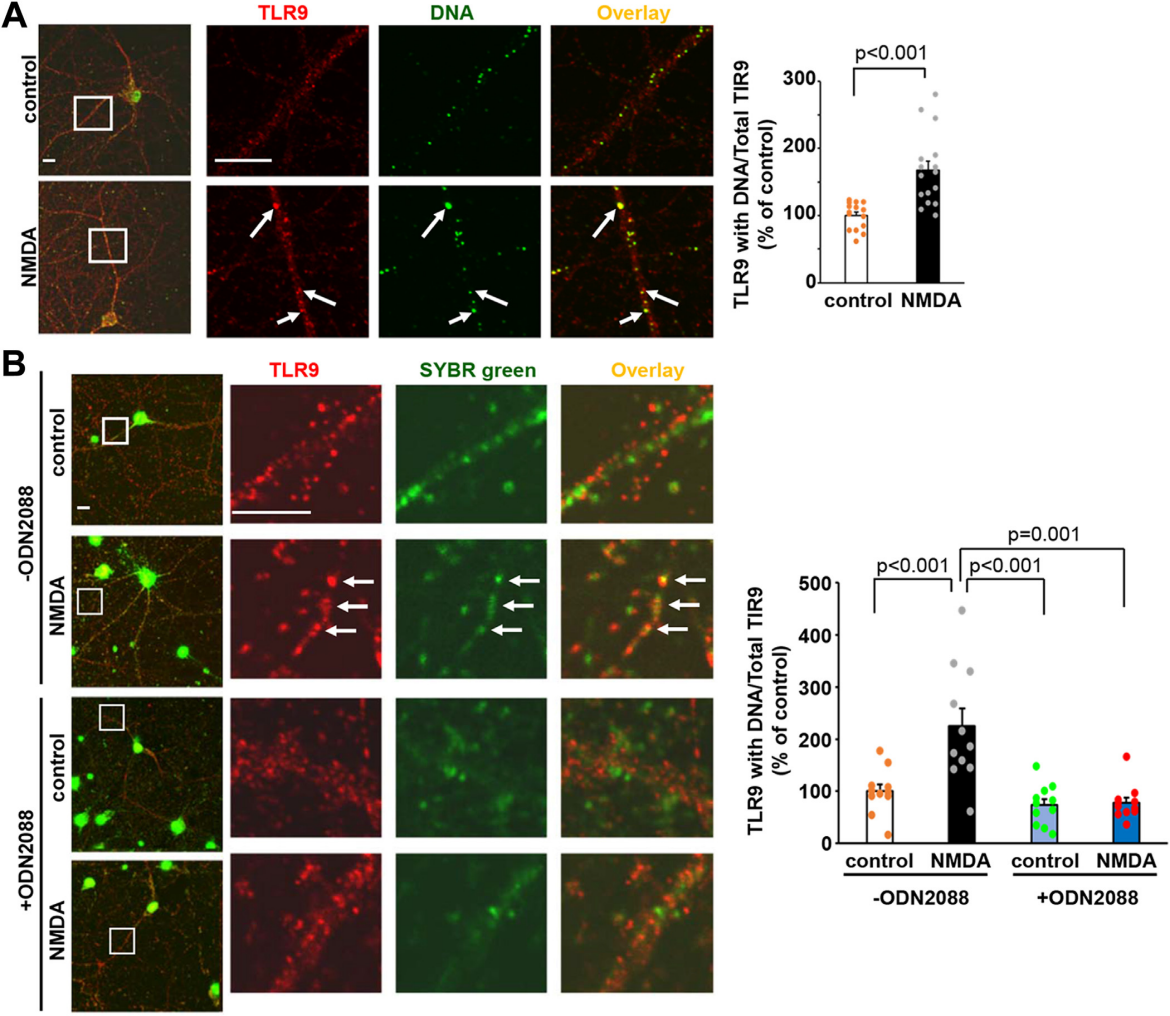
**The interaction between mitochondrial DNA and TLR9 was induced by the NMDA stimulation.** Colocalization of TLR9 with DNA with or without NMDA treatment. *A*, cultured hippocampal neurons were stimulated with NMDA. After fixation, TLR9 and DNA were stained with antibodies. *Arrows* indicate the colocalization of TLR9 and DNA. The scale bars represent 10 μm. *Right graph*: quantification of the colocalization of TLR9 with DNA. Data are represented as the ratio of colocalized TLR9 staining to total TLR9 staining intensity. The ratio in the neurons without NMDA stimulation (control) was defined as 100% (n = 15–16 cells). Data are presented as mean + SEM and individual data points. *p* value, two-tailed Student’s *t* test. *B*, cultured hippocampal neurons were prestained by SYBR green and stimulated with NMDA in the absence or presence of ODN2088 treatment. After fixation, neurons were stained with anti-TLR9 antibody. *Arrows* indicate the colocalization of TLR9 and DNA. The scale bars represent 10 μm. *Right graph*: quantification of the colocalization of TLR9 with DNA. Data are represented as the ratio of colocalized TLR9 staining to total TLR9 staining intensity. The ratio in the ODN2088 untreated control neurons was defined as 100% (n = 11 cells). Data are presented as mean + SEM and individual data points. *p* value by Kruskal–Wallis test and Student-Newman-Keuls post hoc test. NMDA, N-methyl-d-aspartate; TLR, toll-like receptor.

To examine whether mtDNA is associated with TLR9, the intracellular distribution of mtDNA and endogenous TLR9 was analyzed using immunocytochemical analysis. Cultured hippocampal neurons were treated with NMDA for 10 min and TLR9 and DNA were stained with anti-TLR9 and anti-DNA antibodies (Fig. 5*A*). The amount of TLR9 colocalized with mtDNA significantly increased on NMDA stimulation (*p* < 0.001, n = 15–16 cells from two independent cultures prepared on different days, two-tailed Student’s *t* test).

NMDA stimulation affected the distribution of TLR9, as demonstrated in Figure 5*A*. These observations are consistent with a previous report indicating that TLR9 exists in the endoplasmic reticulum before activation and is rapidly translocated to endolysosomes upon activation (39).

To further confirm the mtDNA binding to the TLR9 and examine the effects of ODN2088, we prestained DNA with SYBR Green I and applied NMDA stimulation in the absence or presence of ODN2088. The colocalization of DNA and TLR9 significantly increased with NMDA stimulation in the ODN2088 untreated neurons (Fig. 5*B*; *p* < 0.001, n = 11 from two independent cultures prepared on different days, Kruskal–Wallis test and Student-Newman-Keuls post hoc test). However, NMDA-induced colocalization between TLR9 and SYBR Green I stained DNA was not observed in the ODN2088 treated neurons (*p* = 1.00).

These results suggested that the NMDA-dependent mtDNA release activates TLR9 and that the ODN2088 impedes the association between mtDNA and TLR9.

### Activation of caspase-3, but not the cytochrome c release, was induced by the TLR9

**Figure 6:**
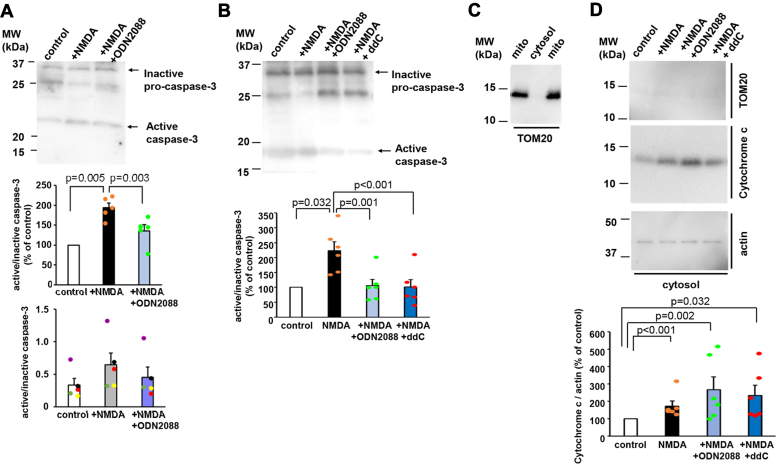
**TLR9 regulates caspase-3 activity but not the release of cytochrome c from mitochondria.***A*, the effect of ODN2088 on the activation of caspase-3. NMDA stimulation increased the ratio of active caspase-3 to inactive procaspase-3 and pretreatment with ODN2088 blocked the NMDA effect. Cultured hippocampal neurons were stimulated with NMDA for 10 min with or without treatment with ODN2088. Neurons were lysed and the activation of caspase-3 was analyzed by immunoblot analysis. The 25 kDa extra band may correspond to the procaspase-3 cleaved only in its N-terminal domain, as previously described (62). *Middle graph*: the intensity of the band corresponding to active caspase-3 was normalized to that of inactive procaspase-3. The ratio of active/inactive caspase-3 in control cultures was arbitrarily set to 100%. This quantification was also used previously by Shen *et al.* (15). Data are presented as mean + SEM and individual data points. *p* value by Kruskal–Wallis test and Student-Newman-Keuls post hoc test. *Lower graph*: the ratio of active/inactive caspase-3 was directly plotted. The same colors of the individual points indicate the data from the same sets of immunoblot analysis. *B*, the effect of ODN2088 and ddC on the prolonged activation of caspase-3. Cultured hippocampal neurons were stimulated with NMDA with or without treatment with ODN2088 and ddC. Neurons were lysed at 30 min after NMDA stimulation and the activation of caspase-3 was analyzed. *Lower graph*: the intensity of the band corresponding to active caspase-3 was normalized to that of inactive procaspase-3. The ratio of active/inactive caspase-3 in the control cultures was arbitrarily set to 100%. Data are presented as mean + SEM and individual data points. *p* value by Kruskal–Wallis test and Student-Newman-Keuls post hoc test, n = 6. *C*, purification of mitochondrial and cytosol fractions from cultured hippocampal neurons. Purified mitochondrial fraction (mito) and cytosol fraction (cytosol) were analyzed by immunoblot analysis using anti-TOM20, a mitochondrial membrane protein, antibody. *D*, effect of ODN2088 and ddC on the cytochrome c release from mitochondria. Cultured hippocampal neurons were stimulated with NMDA with or without treatment with ODN2088 and ddC. Cytosol fractions were prepared at 20 min after stimulation, and analyzed by immunoblot analysis using anti-TOM20, cytochrome c, and actin antibodies. *Lower graph*: the intensity of the band corresponding to cytochrome c was normalized to that of actin. The ratio of cytochrome c/actin in the control cultures was arbitrarily set to 100%. Data are presented as mean + SEM and individual data points. *p* value by one-way ANOVA and Student-Newman-Keuls post hoc test, n = 6. ddC, dideoxycytidine; NMDA, N-methyl-d-aspartate; TLR, toll-like receptor.

To identify downstream proteins of TLR9 that induce LTD, we examined whether ODN2088 also inhibits NMDA-induced caspase-3 activation. This exploration is motivated by previous reports of TLR9 activation inducing caspase-3 activation (19) which is known to play an essential role in LTD induction (11). Following a 10-min NMDA treatment in the presence or absence of ODN2088, hippocampal cultures were lysed and subjected to immunoblot analysis using an anticaspase-3 antibody. The band intensities of activated caspase-3 and inactive procaspase-3 were quantified (Fig. 6*A*). Our findings revealed that the ratio of active caspase-3 to inactive procaspase-3 significantly increased after 10 min of NMDA treatment (control 100%, NMDA 194 ± 12%, n = 5, *p* = 0.005, Kruskal–Wallis test and Student-Newman-Keuls post hoc test.). However, this NMDA-induced effect was negated by pretreatment with ODN2088 (136 ± 16%, n = 5, *p* = 0.003 *versus* NMDA and *p* = 0.095 *versus* control). A direct plot of active/inactive caspase-3 is presented in the lower graph. These results indicate that TLR9 activation is necessary for caspase-3 activation during chemical LTD in hippocampal neurons.

Given that previous research suggested that caspase-3 activation peaks at 30 min after NMDA stimulation in cortical neuronal cultures (11), we examined the ratio of active/inactive caspase-3 at this time point in hippocampal cultures (Fig. 6*B*). We found a significant increase in the ratio of active caspase-3 to inactive procaspase-3 at 30 min after NMDA stimulation (control 100%, NMDA 223 ± 30%, n = 6, *p* = 0.032, Kruskal–Wallis test and Student-Newman-Keuls post hoc test.). This NMDA-induced effect was canceled by the pretreatment with ODN2088 105 ± 21%, n = 6, *p* < 0.001 *versus* NMDA and *p* = 0.810 *versus* control) and ddC (101 ± 25%, n = 6, *p* < 0.001 *versus* NMDA and *p* = 0.961 *versus* control). These results indicated that prolonged activation of caspase-3 occurred, following NMDA stimulation in cultured hippocampal neurons. The NMDA effects were completely blocked not only by ODN2088 but also by the ddC pretreatment (Fig. 6*B*). The percentage of active caspase at 30 min after NMDA treatment (223 ± 30% of control) was tended to be higher than that observed at 10 min after NMDA stimulation (194 ± 12%), although the difference was not significant (*p* = 0.391, n = 5–6, two-tailed Student’s *t* test). According to the previous report (11), caspase-3 activation peaks at 30 min after NMDA stimulation in cortical neurons, which may be consistent with our results from hippocampal neurons.

We also examined whether the release of cytochrome c could be observed at 20 min after NMDA stimulation, as the previous report indicated that the cytochrome c release peaks at 20 min after NMDA stimulation in cortical neuronal cultures (11). For this purpose, we isolated mitochondria and cytosol fractions by using a mitochondria purification kit. Immunoblot analysis with an anti-TOM20 antibody, a mitochondrial resident protein, confirmed the absence of mitochondrial contamination in the cytosol fraction (Fig. 6*C*). We prepared cytosol fractions from control cultures, NMDA stimulated cultures, cultures treated with ODN and NMDA, and cultures pretreated with ddC and stimulated with NMDA, and were analyzed by immunoblot analysis using anti-TOM 20, cytochrome c, and actin antibodies (Fig. 6*D*). The results indicated that the ratio of cytochrome c to actin staining intensities significantly increased upon NMDA stimulation (*p* < 0.001 n = 6, Kruskal–Wallis test and Student-Newman-Keuls post hoc test) and neither ODN2088 nor ddC treatment reduced this ratio (*p* = 0.002 and 0.032 *versus* control). These results indicated that the mtDNA-dependent activation of TLR9 is required for the activation of caspase-3 but not for cytochrome c release.

## Discussion

**Figure 7:**
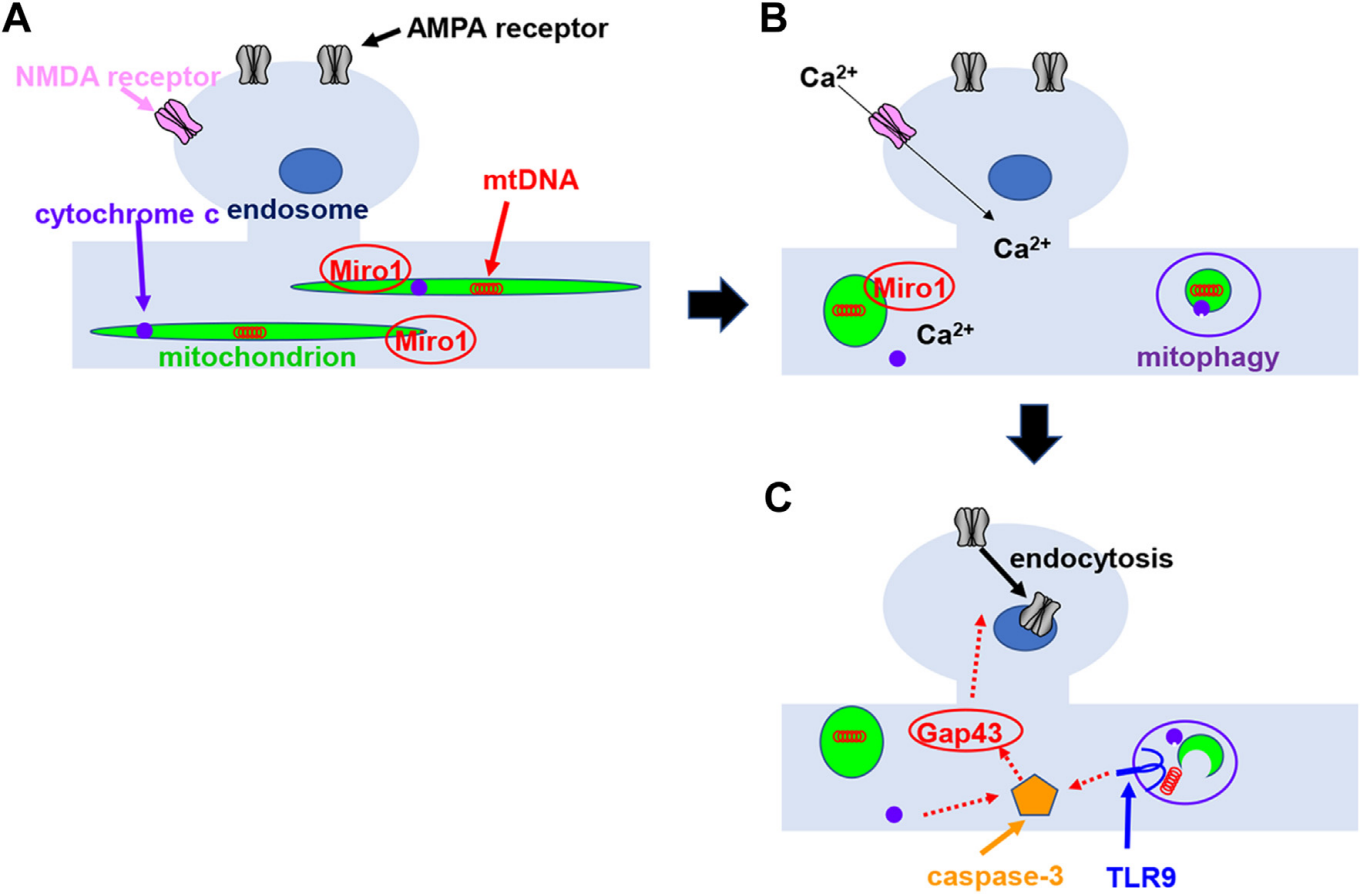
**Model for the mtDNA-TLR9 regulated AMPA receptor trafficking during LTD.***A*, at the basal stage dendritic mitochondria are elongated with mtDNAs stored inside. *B*, due to LTD-inducing Ca^2+^ influx from NMDA receptors, mitochondrial morphology rapidly changes probably mediated by Miro-1, leading to the cytochrome c release and mitophagy. *C*, mtDNAs are released from the mitochondria by mitophagy and activate TLR9. Activated TLR9 and released cytochrome c induce the nonapoptotic activation of caspase-3, which cleaves Gap43 to internalize the AMPA receptors. AMPA, α-amino-3-hydroxy-5-methyl-4-isoxazole propionic acid; LTP, long-term potentiation; Miro-1, mitochondrial Rho GTPase-1; mtDNA, mitochondrial DNA; NMDA, N-methyl-d-aspartateTLR, toll-like receptor.

In our study, we found that TLR9 activation was essential for NMDA-induced endocytosis of AMPA receptors and that NMDA stimulation induced mitophagy and mtDNA association with TLR9. Based on these findings, we propose the following model of TLR9 function in LTD induction (Fig. 7): NMDA receptor activation induces mitochondrial morphological changes *via* elevation of cytoplasmic Ca^2+^ concentration. Previous studies have revealed that mitochondrial Rho GTPase-1 induces mitochondrial shape transition in a cytoplasmic Ca^2+^-dependent manner, which plays a critical role in mitophagy (40). Therefore, mitochondrial Rho GTPase-1 may contribute to LTD induction by inducing mitophagy. Mitophagy releases mtDNA, which associates with TLR9 and may activate TLR9. It is also known that cytochrome c release from mitochondria is required for the activation of caspase-3 (11). Both TLR9 activity and cytochrome c release seem to be required for the activation of caspase-3 to induce LTD.

Furthermore, the downstream targets of caspase-3 have been also identified (41). It has been reported that one of the substrates of active caspase-3 is Gap43 and that the mutant Gap43 which is not cleaved by caspase-3 inhibits the induction of LTD. Activated caspase-3 is thought to digest Gap43, leading to the endocytosis of AMPA receptors during LTD induction. This is the first study to demonstrate that innate immune receptor contributes to synaptic plasticity.

### Nonapoptotic activation of caspase-3 for LTD induction

Our results indicated that mitophagy and TLR9 play essential roles in the caspase-3 activation during the induction of LTD. Previously, it was reported that the rapid but partial activation of caspase-3 by cytochrome c, released from mitochondria, plays essential roles in the induction of LTD without inducing apoptosis (11). The process of how TLR9 and cytochrome c release activate caspase-3 during LTD induction remains unclear. However, it was shown that TLR9 triggers p38 MAPK in the apoptotic signal cascade (23). Moreover, p38 MAPK has been reported to be necessary for the induction of LTD (24). During LTD induction, p38 MAPK may be involved downstream of TLR9.

In the apoptosis pathway, activated p38 MAPK induces the translocation of BAX to mitochondria and the release of cytochrome c from mitochondria (25) to induce cell death. However, the translocation of BAX to mitochondria was not detected during the LTD induction in previous studies (10), and our analysis indicated that the cytochrome c release was not blocked by ODN2088 or ddC treatment. Therefore, cytochrome c release from mitochondria during LTD induction in hippocampal neurons may not be induced by the TLR9-dependent pathway but rather by an unknown pathway. It is also known that p38 MAPK activates caspase-3 by reducing the interaction between caspase-2 and procaspase-3 (26). TLR9 may activate caspase-3 *via* the p38 MAPK-caspase-2 pathways during LTD induction. Indeed, caspase-2 has been found to be important for the internalization of AMPA receptors during LTD induction (27). It is possible that the TLR9 and cytochrome c pathways work together to induce LTD.

### Function of TLR9 and mitophagy in learning and memory

From the study of developing mouse brains, it was shown that the neuronal expression of TLR9 is upregulated on postnatal day 0 and remains high 5 months later (20); however, its function in adult neurons is unknown. The present study elucidates the function of TLR9 in LTD, a form of synaptic plasticity, suggesting that TLR9 regulates some forms of memory and learning (3). For example, hippocampal LTD is required for the consolidation of spatial and fear memories (42, 43). Therefore, TLR9 activation may play an essential role in this process.

Although glucocorticoids, the stress hormone, have been broadly accepted as neuromodulators of memory processes (44), the precise molecular mechanism by which they control synaptic plasticity remains unclear. Recently, they were reported to inhibit mitophagy and reduce spatial learning ability (45). It is also known that the glucocorticoids affect the intracellular trafficking of AMPA receptors (46). In particular, the treatment with corticosterone, a glucocorticoid, increases the number of AMPA receptors on the cell surface of cortical pyramidal neurons (47). These findings suggest that the inhibition of mitophagy by stress hormones affects spatial learning by regulating the intracellular trafficking of AMPA receptors. TLR9 activation through mitophagy, may contribute to the stress hormone–dependent modulation of memory processes.

### Pathological AMPA receptor endocytosis and TLR9

Physiological LTD is induced in a temporally and spatially tightly controlled manner. In contrast, uncontrolled endocytosis of AMPA receptors can be induced by treatment with amyloid β, which does not require synaptic NMDA activation. This uncontrolled endocytosis has been linked to memory loss (48, 49). Since amyloid β has also been shown to activate mitophagy (50), amyloid β may induce the activation of TLR9 and enhance the endocytosis of AMPA receptors without synaptic NMDA activation.

Moreover, since TLR9 can be activated by the viral DNA (51) as well as the mtDNA, viral infection may also induce uncontrolled endocytosis of AMPA receptors. For example, TLR9 recognizes herpes simplex virus type 1 (HSV-1) (52) and type 2 (HSV-2) (53). Interestingly, HSV-1 has been proposed as a possible etiological factor in Alzheimer’s disease (54). Moreover, AZP2006, which has a protective effect on neurons injured by amyloid β, inhibits TLR9 (55). TLR9-dependent trafficking of AMPA receptors may contribute to Alzheimer's disease.

TLR9 activation has also been reported to be induced by severe acute respiratory syndrome coronavirus 2 (coronavirus infectious disease, emerged in 2019) infection *via* mtDNA (56). It has been reported that there is a risk of memory disorders in the postacute phase of coronavirus infectious disease, emerged in 2019 (57).

mtDNA-TLR9–dependent trafficking of AMPA receptors may be one of the etiologies of memory disorders. Further studies are warranted to clarify the physiological and pathological functions of TLR9-dependent AMPA receptor trafficking.

## Experimental procedures

### Mice

All procedures related to animal care and treatment were performed in accordance with the guidelines approved by the animal resource committees of the University of Electro-communications. Mice were housed under a 12:12 h light-dark cycle with food and water available *ad libitum*.

### Chemicals and antibodies

NMDA was purchased from Tocris Bioscience. EZ-link sulfo-NHS biotin and SYBR Green 1 from Thermo Fisher Scientific and ODN2088 from InvivoGen. ddC and mission predesigned siRNAs for TLR3 (SASI_Mm01_0018762 and SASI_Mm01_0018762) and TLR7 (SASI_Mm01_00199946 and SASI_Mm01_00199945), TLR9 (SASI_Mm01_00126149 and SASI_Mm01_00126147) were purchased from Sigma-Aldrich. Scramble RNAs for TLR9 siRNA were synthesized and purchased from Eurofins genomics. Commercial antibodies: anti-HA, (Covance 901501), anti-actin (Sigma-Aldrich A3853), anti-GluA2 N terminus (Millipore MAB397), anti-GluA2 C terminus (Millipore AB10529), anti-FLAG (Sigma F7425), anti-caspase-3 (ProteinTech 66470-2-Ig), anti-TLR9 (Abcam ab134368), anti-cytochrome c ProteinTech 10993-1-AP), anti-TOM20 (ProteinTech 11802-1-AP) and anti-DNA (Millipore CBL186) antibodies; Alexa-350 (Thermo Fisher Scientific A-11045), 405 (Thermo Fisher Scientific A-31556), 488 (Thermo Fisher Scientific A-11008), 546 (Thermo Fisher Scientific A-11003), horseradish peroxidase (Rockland 18-8816-33, 18-8817-33)-conjugated secondary antibodies.

Antibodies used for the immunocytochemical analysis have been validated in antigen-overexpressed cells or the siRNA-mediated antigen knocked down cells. The specificity of antibodies used in immunoblot analysis has been verified by the detection of proteins with appropriate molecular weight. The specificity of secondary antibodies was confirmed by the omission of primary antibodies.

### Construction and transfection of expression plasmids

Using PCR and Pyrobest (Takara) or KOD One (Toyobo), the cDNA encoding HA was added to the 5′ end (immediately following the signal sequence) of WT GluA2 (HA-GluA2). The cDNA encoding the FLAG-tag was added to the 3′ end (immediately upstream of the stop codon) of mouse TLR9 cDNAs (TLR9-Flag). cDNA encoding mCherry was added to the 5′ end of mouse LC3B (mCherry-LC3B). The mito-CFP was obtained from Clontech. Nucleotide sequences of the amplified ORFs were confirmed by bidirectional sequencing. After the cDNAs were cloned into the expression vectors, pCAGGS (provided by Dr J Miyazaki, Osaka University), the constructs were transfected into HEK293 cells using the Ca^2+^-phosphate method or were transfected into cultured hippocampal neurons using Lipofectamine 2000 (Invitrogen). For cotransfection with siRNA, hippocampal neurons were transfected using TransMessenger (Qiagen).

### Culture of hippocampal neuron

Hippocampi dissected from E17 ICR mice were treated with 10 U ml^−1^ trypsin and 100 U ml^−1^ DNase in Dulbecco's modified Eagle's medium at 37 °C for 20 min. Dissociated hippocampal neurons were plated on PEI-coated glass coverslips and cultured in neurobasal medium (Invitrogen) with B-27 supplement (Gibco) and 0.5 mM L-glutamine. After 7 days *in vitro* (DIV) culture, neurons were transiently transfected with plasmids using Lipofectamine 2000 or siRNA and plasmids with TransMessenger and were used for AMPA receptor endocytosis or immunoblot analysis. For TLR9 inhibitor treatment, cultured hippocampal neurons were pretreated with ODN2088 at a final concentration of 1 μM for 10 min. For ddC pretreatment, neurons were treated with ddC at a final concentration of 10–20 μM for 96 h (replenished every 48 h), as described by West *et al.* (38). For Mdivi-1 pretreatment, neurons were treated with Mdivi-1 at a final concentration of 10 μM for 2 h as described by Liu *et al.* (37).

### Assay for AMPA receptor endocytosis

Hippocampal neurons transfected with pCAGGS expression vectors for HA-GluA2 or with siRNA and expression vector for HA-GluA2 (plus mouse TLR9-Flag carrying a siRNA-resistant mutation) were stimulated with 50 μM NMDA for 10 min and fixed in 4% paraformaldehyde without permeabilization for 10 min at room temperature. After washing with PBS and incubation with a blocking solution (2% bovine serum albumin [BSA] and 2% normal goat serum in PBS), surface HA-GluA2 was labeled with the anti-HA antibody (1/1000) and visualized with Alexa 546–conjugated secondary antibody (1/1000). To label total HA-GluA2, neurons were permeabilized and blocked with a blocking solution containing 0.4% Triton X-100 and incubated with anti-HA antibody (1/1000) and Alexa 350–conjugated secondary antibodies (1/1000). Fluorescence images were captured using a fluorescence microscope (BX60, Olympus) equipped with a charge-coupled device camera (DP 70, Olympus) and analyzed using the IP-Lab software (Scanalytics; https://www.digitalimagingsystems.co.uk/pdfs/IPLab_VIEW-me.pdf). For statistical analysis of the surface expression level of HA-GluA2, the intensity of Alexa 546 for surface HA-GluA2 was measured and normalized using the intensity of Alexa 350 for total HA-GluA2. The fluorescence intensity on dendrites at a distance of at least 20 μm away from the soma was measured. In the representative images, brightness and contrast were adjusted uniformly within each experimental series for consistent visibility.

### Chemical LTP in hippocampal neurons

Cultured hippocampal neurons were transfected with plasmids encoding HA-GluA1, At DIV 17, the cultured medium was changed to an extracellular solution with the following composition (in mM): NaCl, 140; CaCl_2_, 1.3; KCl, 5.0; Hepes, 25; glucose, 33; TTX, 0.0005; strychnine, 0.001; and bicuculline methiodide, 0.02 (pH 7.4), as described by Lu *et al.* (28). Neurons were stimulated with 200 μM glycine for 3 min and transferred to the external solution without glycine. After 17 min of further incubations, neurons were fixed in 4% paraformaldehyde without permeabilization for 10 min at room temperature and incubated with a blocking solution containing 2% BSA and 2% normal goat serum in PBS. Cell surface HA-GluA1 was labeled with the anti-HA antibody (1:1000) and visualized with Alexa546 secondary antibody (1:1000). Cells were then permeabilized with 0.4% Triton X-100 in the blocking solution and total HA-GluA1 was labeled with the anti-HA (1:1000) and Alexa350 secondary antibodies (1:1000). Fluorescence images were captured using a fluorescence microscope (BX60, Olympus) equipped with a charge-coupled device camera (DP70, Olympus) and analyzed using IP-Lab software (Scanalytics). For statistical analysis of the surface expression level of HA-GluA1, the intensity of Alexa546 for surface HA-GluA1 was measured and normalized using the intensity of Alexa350 for total HA-GluA1. The fluorescence intensity on dendrites at least 20 μm away from the soma was measured. In the representative images, brightness and contrast were adjusted uniformly within each experimental series for consistent visibility.

### Cell surface biotinylation assay

Cell surface biotinylation was conducted as described by Azarnia Tehran *et al.* (58). Briefly, cultured hippocampal neurons with or without NMDA stimulation were washed once with ice-cold PBS^2+^ (comprising 137 mM NaCl, 2.7 mM KCl, 10 mM Na_2_HPO_4_, 1.8 mM KH_2_PO_4_, 1 mM CaCl_2_, and 0.5 mM MgCl_2_ [pH 7.4]) and incubated with the EZ-link sulfo-NHS biotin for 20 min at 4 °C. After quenching with 50 mM glycine, neurons were solubilized in radio-immunoprecipitation assay buffer. After centrifugation at 13000*g* for 10 min, supernatants were used as the total cell lysate fractions. Cell surface biotinylated proteins were pulled down by streptavidin beads. Total lysates and pulled down fractions were analyzed by immunoblot analysis using an anti-GluA2 C-terminal antibody. Chemiluminescence signals were detected by Luminograph II (ATTO) and quantified using the CS Analyzer software (ATTO; https://www.attoeng.site/cs-analyzer4).

### Antibody feeding assay

Antibody feeding assay was conducted following the procedure described by Li *et al.* (11) and Azarnia Tehran *et al.* (58). Briefly, hippocampal neurons transfected with pCAGGS expression vectors for HA-GluA2 and untransfected neurons were incubated with the antibodies against HA and the N terminus of GluA2, respectively (20 μg/ml), for 15 min at 37 °C. Neurons were fixed after the NMDA stimulation, and the surface-remaining antibodies were stained with Alexa 546–conjugated secondary antibody (1/1000). To label internalized antibodies, neurons were permeabilized and blocked with a blocking solution containing 0.4% Triton X-100 and incubated with Alexa 488–conjugated secondary antibodies (1/1000).

### Analysis of siRNA knockdown effects in HEK tSA cells

HEK 293tSA cells (59, 60) were transfected with plasmids that encode TLR9-Flag and TLR9 siRNA in φ6 cm dishes. Two days after transfection, cells were solubilized in 300 μl of Tris NP40 EDTA buffer (50 mM NaCl, 10% NP-40, 20 mM EDTA, 0.1% SDS, 50 mM Tris–HCl, pH 8.0) supplemented with a protease inhibitor cocktail (Calbiochem). Further, 10 μl of total lysate was subjected to immunoblot analysis with anti-FLAG (1/500) (Sigma) antibody. In the representative images, brightness and contrast were adjusted uniformly within each experimental series for consistent visibility. HEK293tSA cells were routinely tested negative for *mycoplasma* contamination.

### Analysis of mitochondrial morphology and mitophagy

Hippocampal neurons were transfected with pCAGGS expression vectors for mito-CFP plus mCherry or mCherry-LC3B. For live imaging, neurons were maintained in a stage-top incubator (STR, TOKAI HIT) on a confocal microscope (FV1200, Olympus). CFP and mCherry fluorescence were captured every minute before and after 50 μM NMDA application in the presence or absence of ODN2088 pretreatment (1 μM for 10 min). For the analysis of fixed neurons, cultures were fixed using the method described by Kim *et al.* to prevent mitochondrial fragmentation (61).

### Colocalization assay of TLR9 and DNA

Hippocampal neurons with or without SYBR Green I prestaining (1/2000 for 30 min at 37 °C) were stimulated with 50 μM NMDA for 10 min with or without ODN2088 pretreatment and fixed in 4% paraformaldehyde. After the fixed neurons were washed with PBS and incubated with a blocking solution (2% BSA, 2% normal goat serum, and 0.4% Triton X-100 in PBS), the neurons were incubated with anti-TLR9 antibody (1/1000) and for SYBR Green I-unstained neurons, anti-DNA antibody (1/250) for 1 h at room temperature. After washing with PBS, the neurons were incubated with Alexa 546– and, for SYBR Green I–unstained neurons, with Alexa 488–conjugated secondary antibodies (1/1000; Invitrogen). Fluorescence images were captured using a confocal microscope (FV1200, Olympus). To statistically analyze the colocalization of TLR9 with DNA, the intensity of Alexa 546 on Alexa 488–positive regions was measured and normalized using the total intensity of Alexa 546. This completed using an IP-Lab software (Scanalytics) by a second investigator, who was blinded to the experimental conditions. The blind was not broken until data analysis was complete. The fluorescence intensity on the dendrites, at least 20 μm away from the soma, was measured.

### Caspase-3 activation assays

Cultured hippocampal neurons at DIV 17 from three wells of 12-well dishes (Falcon) with or without NMDA treatment (50 μM, 10 min) or neurons incubated for 30 min after NMDA stimulation were solubilized in 300 μl of 2X SDS sample buffer containing 50 mM NaCl, 20% glycerol, 200 mM DTT, 4% SDS, 0.2% bromophenol blue, and 125 mM Tris–HCl, pH 6.8. Further, 10 μl of total lysate was subjected to immunoblot analysis using anticaspase-3 (1/1000) (ProteinTech) antibody, TrueBlot horseradish peroxidase–conjugated secondary antibody (1/1000) (Rockland), and immobilon Western kit (Millipore). Chemiluminescence signals were detected by Luminograph II (ATTO) and quantified using the CS Analyzer software (ATTO). In the representative images, brightness and contrast were adjusted uniformly within each experimental series for consistent visibility.

### Purification of mitochondrial and cytosol fraction

Mitochondrial and cytosol fractions were purified from cultured hippocampal neurons using a mitochondria isolation kit for cultured cells (89874: Thermo Fisher Scientific) according to the manufacturer's protocol. Purified mitochondrial and cytosol fractions were analyzed by immunoblot analysis using anti-TOM20, cytochrome c, and actin antibodies.

### Statistical analysis

Data are presented as the mean ± SEMs or mean + SEM. All tests were performed with Sigma Plot (Systat Software Inc; https://grafiti.com). Data were tested for normal distribution by the Shapiro–Wilk normality test. For the comparison between two groups, we used two-tailed Student’s *t* test. For comparison among several groups, ANOVA or Kruskal–Wallis test, followed by post hoc tests, were used. A *p*-value of <0.05 was considered statistically significant.

## Data availability

All the data described are presented either within the article or in the supporting information.

## Supporting information

This article contains supporting information.

## Conflict of interest

The authors declare that they have no conflicts of interest with the contents of this article.
