# Supplementary material for: Activation of innate immune receptor TLR9 by mitochondrial DNA plays essential roles in the chemical long-term depression of hippocampal neurons
Source: J Biol Chem. 2024 Feb 13;300(3):105744. doi: 10.1016/j.jbc.2024.105744 (PMC10943477; doi:10.1016/j.jbc.2024.105744)
Supplement: Supplemental Figs. S1–S7 [file mmc2.docx]

Supporting Information

Activation of innate immune receptor TLR9 by mitochondrial DNA plays essential roles in the chemical long-term depression of hippocampal neurons

Naoya Atarashi^1*^, Misaki Morishita^1*^, Shinji Matsuda^1,2^

1Department of Engineering Science, Graduate School of Informatics and Engineering; 2Center for Neuroscience and Biomedical Engineering (CNBE), The University of Electro-Communications, Tokyo 182-8585, Japan

*These authors contributed equally to the work.

Table of contents:

Movie S1: NMDA-induced morphological changes of mitochondria and dendrites

Figure S1: ODN2088 blocks NMDA-induced reduction but not glycine-induced increase in cell surface GluA1

Figure S2: Toll-like receptor (TLR) expression in cultured hippocampal neurons and the siRNA effects on the TLRs expression.

Figure S3: Expression of wild-type TLR9 did not rescue the effect of TLR9 siRNA

Figure S4: Mitochondrial morphological changes and mitophagy induced by NMDA treatment.

Figure S5: Reversibility of the Mitochondrial morphological changes after NMDA treatment

Figure S6: Mdivi-1 treatment blocked the mitophagy induced by NMDA treatment.

Figure S7: ddC treatment had no effects on the morphological changes of mitochondria induced by NMDA treatment

**
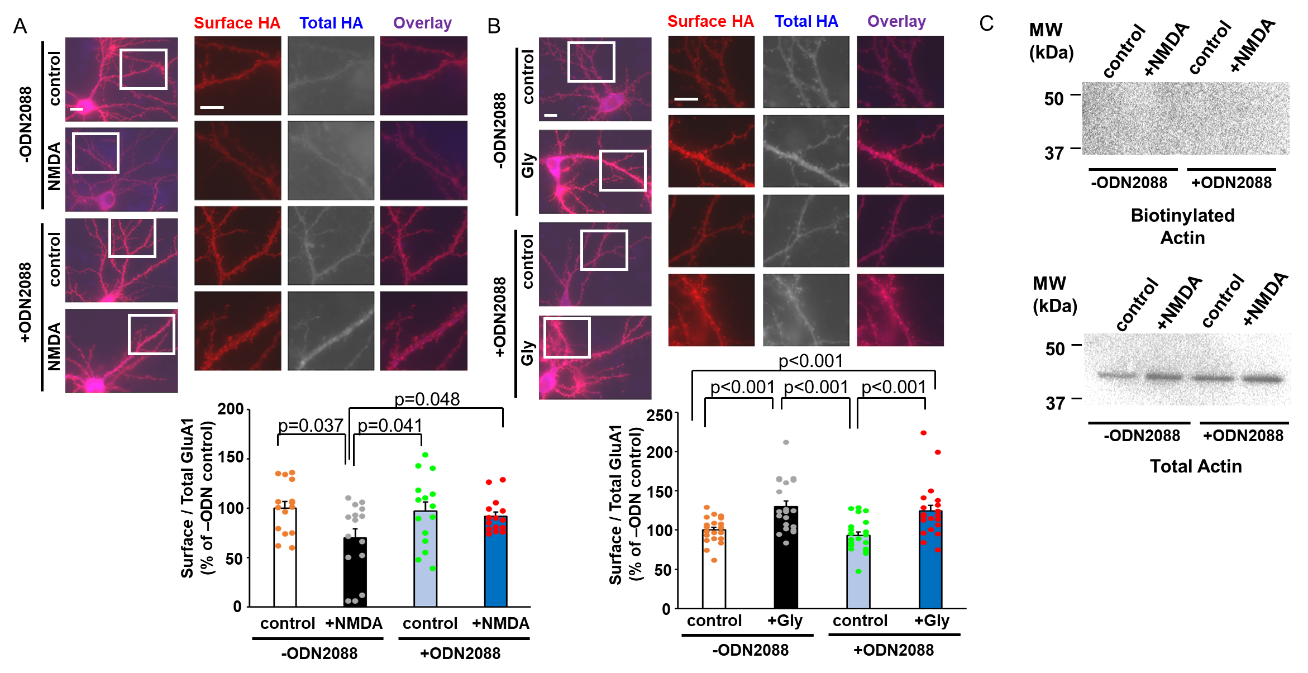
**

**Figure S1. ODN2088 blocks NMDA-induced reduction but not glycine-induced increase in cell surface AMPA receptor.** Cultured hippocampal neurons expressing hemagglutinin (HA)-tagged GluA1 (**A**) were treated with 50 µM NMDA for 10 min without or with ODN2088 (1 μM, 10 min). After fixation, cell surface HA-GluA1 was stained (red) and after treatment with Triton-X, neurons were immunostained for total HA-GluA1 (blue). The dendritic regions marked by squares are enlarged in the panels to the right. Scale bar, 10 µm. (**Lower graph**) Quantification of the NMDA-induced reduction in the ratio of surface to total HA-GluA1 fluorescence intensities. The ratio of control neurons without ODN2088 treatment was defined as 100% (n = 22). Data are presented as mean + standard errors of the mean (SEM) and individual data points. p value by one-way ANOVA followed by Student-Newman-Keuls post hoc test. (**B**) Immunocytochemical analysis of the effects of ODN2088 on the glycine (Gly)-induced elevation of cell surface HA-GluA1. Cultured hippocampal neurons expressing HA-tagged GluA1 were treated with 200 µM Gly without or with ODN2088. After fixation, cell surface HA-GluA1 (red) and total HA-GluA1 (blue) were sequentially stained. The dendritic regions marked by squares are enlarged in the panels to the right. Scale bar, 10 µm. (**Lower graph**) Quantification of the Gly-induced elevation in the ratio of surface to total HA-GluA1 fluorescence intensities. Data are presented as mean + standard errors of the mean (SEM) and individual data points. p value by one-way ANOVA followed by Student-Newman-Keuls post hoc test. (**C**) Biotinylation assay of actin. Hippocampal cultures were stimulated by NMDA without or with ODN2088. Cell surface proteins were biotinylated and pulled down from the total cell lysates. The amount of actin proteins in the pulled-down fraction and total cell lysate fraction were analyzed by the immunoblot analysis.

**
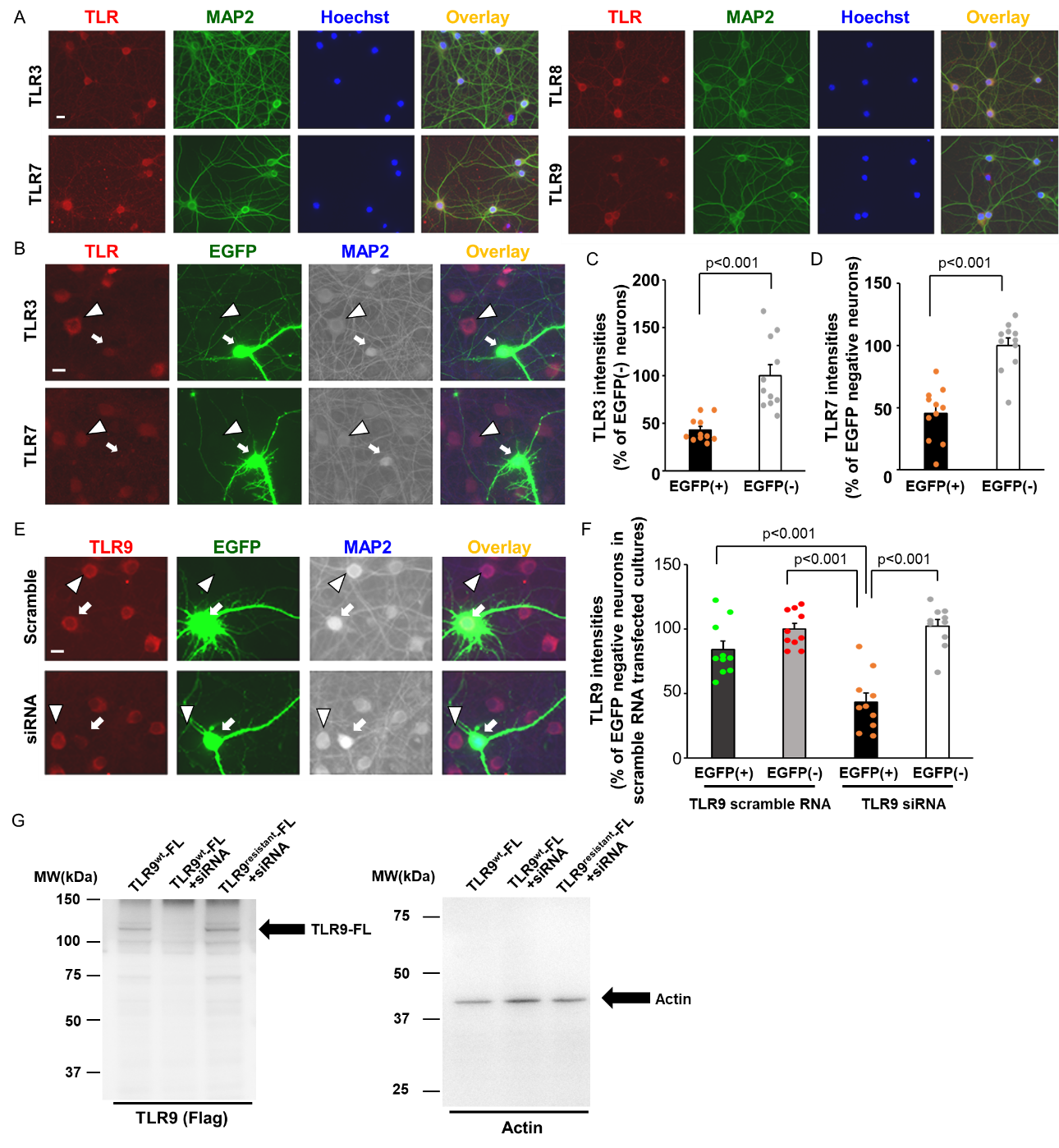
**

**Figure S2. Toll-like receptor (TLR) expression in cultured hippocampal neurons and the siRNA effects on the TLRs expression.** (**A**) Immunocytochemical analysis of the expression of TLRs in hippocampal cultures. Hippocampal cultures were stained by anti-TLR3, TLR7, TLR8, and TLR9 antibodies, MAP2, a neuronal dendritic marker, antibody, and Hoechst. All of TLR3, TLR7, TLR8, and TLR9 proteins were expressed in the hippocampal neurons. Note that majority of the cultured cells are MAP2-positive neurons. Scale bar, 10 µm. (**B**) Immunocytochemical analysis of the effects of siRNA on TLR expression in cultured hippocampal neurons. siRNA for TLR3 (upper panel) or siRNA for TLR7 (lower panel) was transfected into cultured hippocampal neurons with the expression vector for EGFP. Arrows and arrowheads indicate EGFP-positive transfected neurons and neighboring EGFP-negative neurons, respectively. Scale bar, 10 µm. (**C, D**) Quantitative analysis of the fluorescence intensities of TLR3 (**C**) and TLR7 (**D**) immunoreaction in the somatic region of siRNA-transfected (EGFP(+)) and neighboring neurons (EGFP(-)). The average intensity of TLRs in neighboring EGFP-negative neurons was arbitrarily set to 100%. Data are presented as mean + SEM and individual data points. n = 11 from two independent cultures prepared in different days, p value by two-tailed Student’s t-test. (**E**) Immunocytochemical analysis of the effects of siRNA on TLR9 expression in cultured hippocampal neurons. Scramble RNA (upper panel) or siRNA for TLR9 (lower panel) was transfected into cultured hippocampal neurons with the expression vector for EGFP. Arrows and arrowheads indicate EGFP-positive transfected neurons and neighboring EGFP-negative neurons, respectively. Scale bar, 10 µm. (**F**) Quantitative analysis of the fluorescence intensities of TLR9 immunoreaction in the somatic region of siRNA- or scrambled RNA-transfected neurons and neighboring EGFP-negative neurons. The average intensity of TLR9 in the neighboring EGFP negative neurons in the scrambled RNA transfected culture was arbitrarily set to 100%. Data are presented as mean + SEM and individual data points. p value by one-way ANOVA followed by Student-Newman-Keuls post hoc test, n = 10 from two independent cultures prepared in different days. (**G**) Immunoblot analysis of the effects of siRNA and siRNA-resistant mutations. Human embryonic kidney 293 cells were transfected with expression vectors for FLAG-tagged wild-type (TLR9^wt^-FL) or mutant TLR9 carrying resistant mutations for siRNA (TLR9^resistant^-FL) with or without siRNA for TLR9. The arrow indicates FLAG-tagged TLR9 (**left gel**) and endogenous protein, actin (**right gel**). The expression of TLR9-FLAG was almost completely abolished by the co-transfection of siRNA, and the effect of siRNA was rescued by the siRNA-resistant mutation.


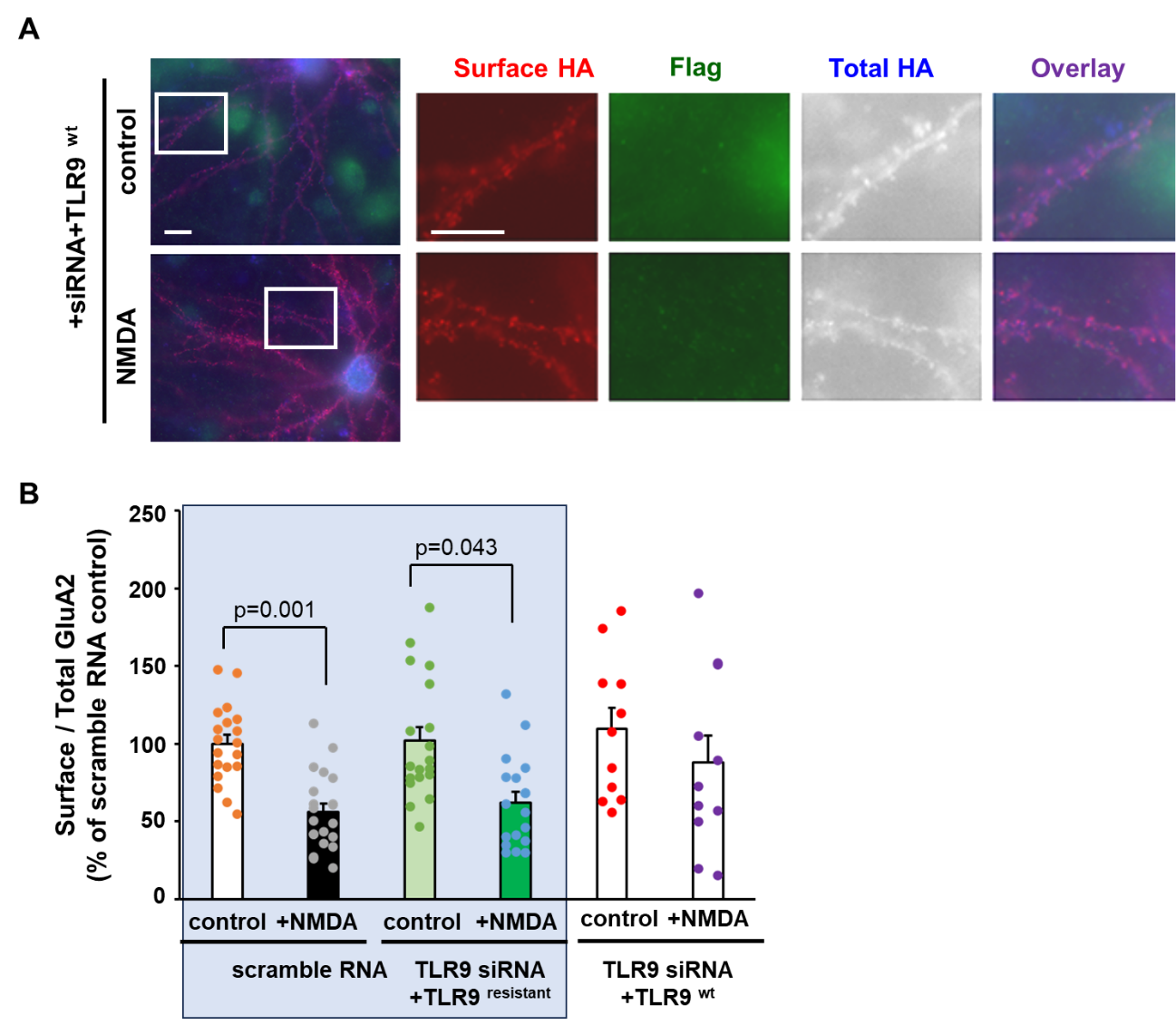


**Figure S3.** **Expression of wild-type TLR9 did not rescue the effect of TLR9 siRNA.** Cultured hippocampal neurons expressing HA-tagged GluA2 and TLR9^wtt^-FL with siRNA treated with 50 µM NMDA for 10 min. (**A**) After fixation, the cell surface HA-GluA2 (red), TLR9^wt^-FL (green), and total HA-GluA2 (blue) were stained. The dendritic regions marked by squares were enlarged in the panels to the right. Scale bar, 10 µm. (**B**) Quantification of NMDA-induced reduction in the ratio of surface to total GluA2 fluorescence intensity. Data are represented as the ratio of surface HA-GluA2 immunoreactivity normalized by total HA-GluA2 immunoreactivity. The bar graphs in the shaded region are identical data to those of Fig. 2C. Data are presented as mean + SEM and individual data points. NMDA stimulation did not induce a significant reduction in the surface/total GluA2 ratio in the TRR9siRNA and wild-type TLR9 expressed neurons (p = 0.347 n = 11 from 2 independent cultures, by two-tailed Student’s t-test).


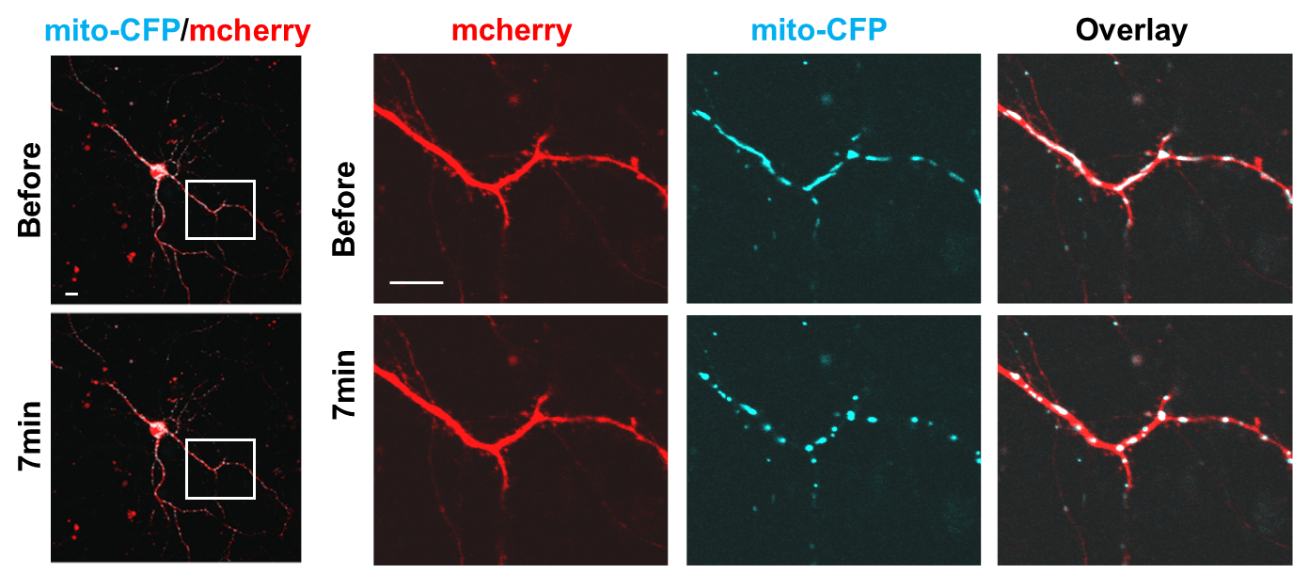


**Figure S4. Mitochondrial morphological changes and mitophagy induced by NMDA treatment.** Cultured hippocampal neurons expressing mitochondria-targeted cyan fluorescent protein (mito-CFP) and mCherry were stimulated with NMDA and observed for up to 7 min (Identical neuron to the Fig. 3A top panel). Images of the mitochondria and dendrites before and 7 min after NMDA treatment. The dendritic region enclosed by the white square is magnified in the right panel. Scale bars represent 10 μm.


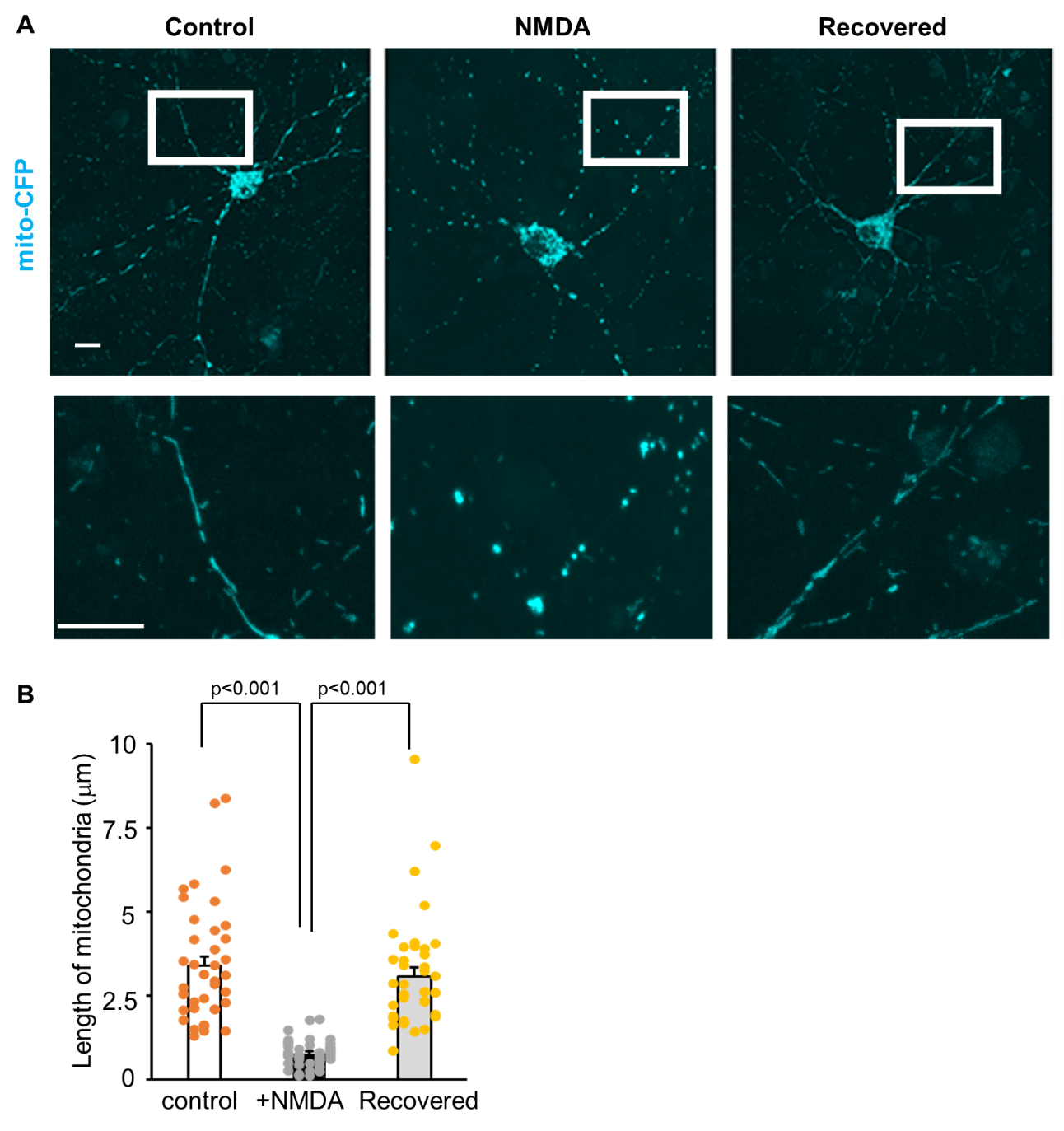


**Figure S5.** **Reversibility of the Mitochondrial morphological changes after NMDA treatment.** Cultured hippocampal neurons expressing mitochondria-targeted cyan fluorescent protein (mito-CFP) were stimulated with NMDA for 7 min and the neurons were washed with Neurobasal medium and incubated for 2 hours. After fixation, the morphology of mitochondria was analyzed. (**A**) Images of the mito-CFP from NMDA untreated (control), NMDA stimulated (NMDA), and 2 hours recovered neurons (Recovered). The dendritic region enclosed by the white square is magnified in the lower panel. Scale bars represent 10 μm. (**B**) Quantitative analysis of the length of mitochondria. n = 37-50 mitochondria from three independent cultures. Data are presented as mean + SEM and individual data points. p value by Kruskal-Wallis test and Dunn’s post-hoc test.


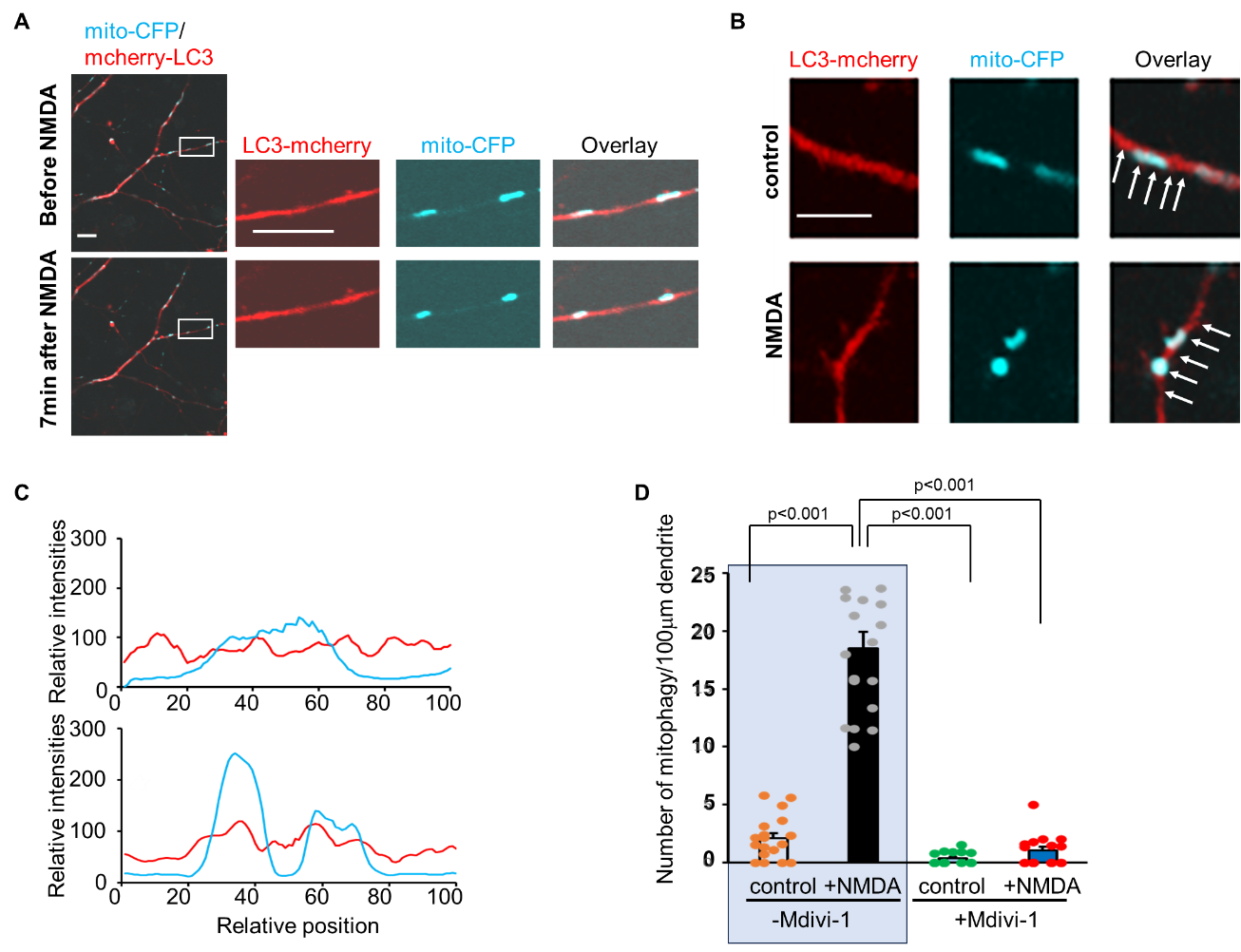


**Figure S6.** **Mdivi-1 treatment blocked the mitophagy induced by NMDA treatment.** (**A**) Cultured hippocampal neurons expressing mitochondria-targeted cyan fluorescent protein (mito-CFP) and mCherry-LC3 were stimulated with NMDA in the presence of Mdivi-1 and observed for up to 7 min. Images of the neurons before and 7 min after NMDA stimulation are shown. The dendritic region enclosed by the white square is magnified in the right panel. Scale bars represent 10 μm. (**B**) High-resolution images of mCherry-LC3 and mito-CFP from the NMDA untreated (control) and NMDA stimulated neurons. Scale bar, 5μm. (**C**) Line scan of the fluorescence intensities of mCherry-LC3 and mito-CFP. The mCherry-LC3 (red) and mito-CFP (cyan) fluorescence were quantified along the dendrites indicated by white arrows in (**B**), indicating that the mito-CFP signal was not surrounded by the mCherry-LC3 signal even in the NMDA-stimulated neuron. (**D**) Quantitative analysis of the number of mitophagy within the 100μm dendrite. The bar graphs in the shaded region are identical data to those of Fig. 3H. Data are presented as mean + SEM and individual data points. n = 18 from three independent cultures. p value by two-tailed Student’s t-test.

**
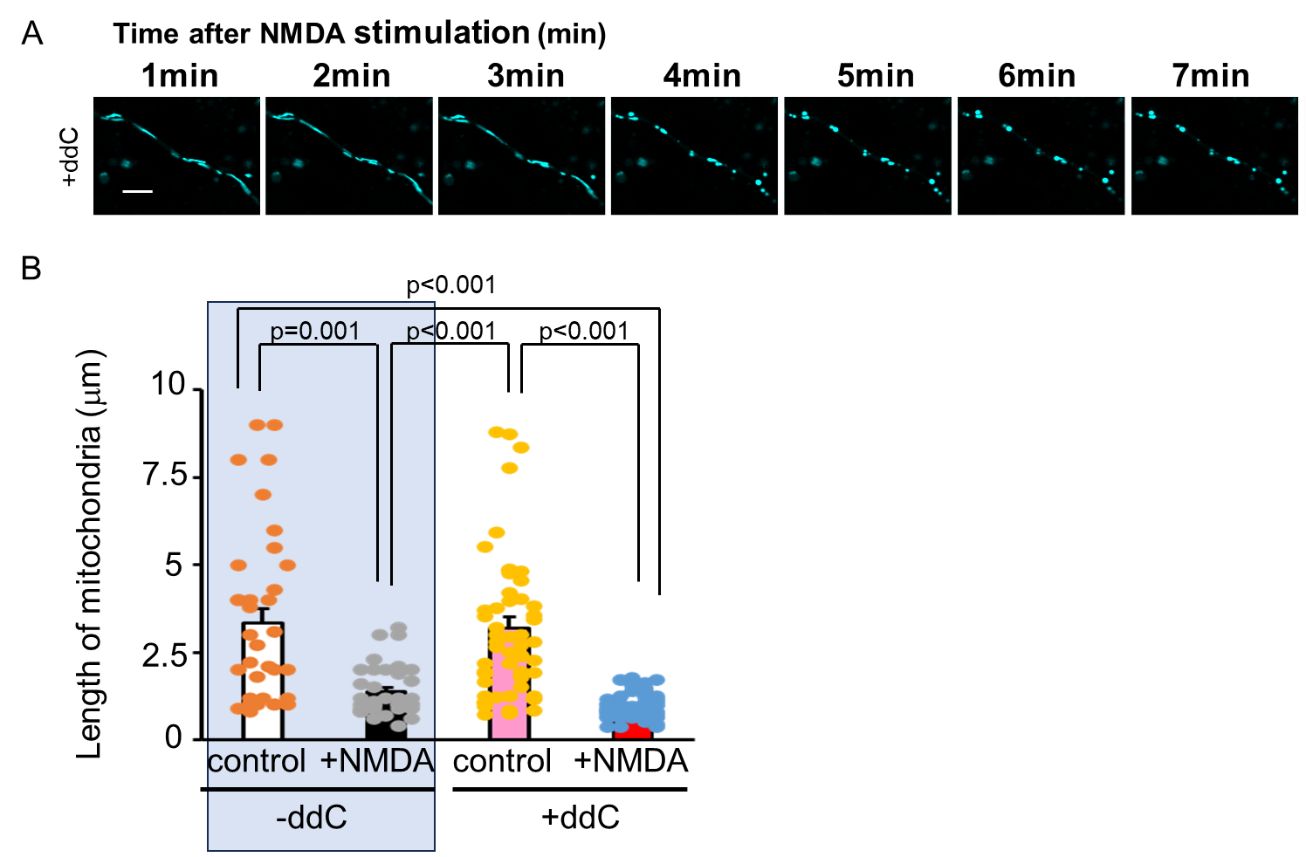
**

**Figure S7.** **ddC treatment had no effects on the morphological changes of mitochondria induced by NMDA treatment.** (**A**) Cultured hippocampal neurons expressing mitochondria-targeted cyan fluorescent protein (mito-CFP) were pretreated by ddC for 96 hours, stimulated with NMDA, and observed for up to 7 min. Images of the mitochondria every 1 min after NMDA stimulation. Scale bar represents 10 μm. (**B**) Quantitative analysis of the length of mitochondria. Cultured hippocampal neurons expressing mito-CFP were pre-treated by ddC for 96 hours and stimulated with NMDA for 7min. After fixation, the length of mitochondria was quantified. n = 37-50 mitochondria from three independent cultures. The bar graphs in the shaded region are identical data to those of Fig. 3B. Data are presented as mean + SEM and individual data points. p value by Kruskal-Wallis test and Dunn’s post-hoc test.
